# Advanced cancer targeting using aptamer functionalized nanocarriers for site-specific cargo delivery

**DOI:** 10.1186/s40824-023-00365-y

**Published:** 2023-05-06

**Authors:** Mahavir Narwade, Aazam Shaikh, Kavita R. Gajbhiye, Prashant Kesharwani, Virendra Gajbhiye

**Affiliations:** 1 Department of Pharmaceutics, Poona College of Pharmacy, Bharati Vidyapeeth, Pune, India; 2 Nanobioscience Group, Agharkar Research Institute, Pune, 411004 India; 3 Savitribai Phule Pune University, Ganeshkhind, Pune, 411 007 India; 4 Department of Pharmaceutics, School of Pharmaceutical Education and Research, Jamia Hamdard, New Delhi, 110062 India; 5 Center for Transdisciplinary Research, Department of Pharmacology, Saveetha Dental College, Saveetha Institute of Medical and Technical Science, Chennai, India

**Keywords:** Aptamers, SELEX, Nanocarriers, Cancer targeting, Nanoconjugates, Receptor-mediated endocytosis

## Abstract

The non-specificity of standard anticancer therapies has profound detrimental consequences in clinical treatment. Therapeutic specificity can be precisely achieved using cutting-edge ligands. Small synthetic oligonucleotide-ligands chosen through Systematic evolution of ligands by exponential enrichment (SELEX) would be an unceasing innovation in using nucleic acids as aptamers, frequently referred to as "chemical antibodies.” Aptamers act as externally controlled switching materials that can attach to various substrates, for example, membrane proteins or nucleic acid structures. Aptamers pose excellent specificity and affinity for target molecules and can be used as medicines to suppress tumor cell growth directly. The creation of aptamer-conjugated nanoconstructs has recently opened up innovative options in cancer therapy that are more effective and target tumor cells with minor toxicity to healthy tissues. This review focuses on a comprehensive description of the most capable classes of aptamer-tethered nanocarriers for precise recognition of cancer cells with significant development in proficiency, selectivity, and targetability for cancer therapy. Existing theranostic applications with the problems and future directions are also highlighted.

**Graphical Abstract:**

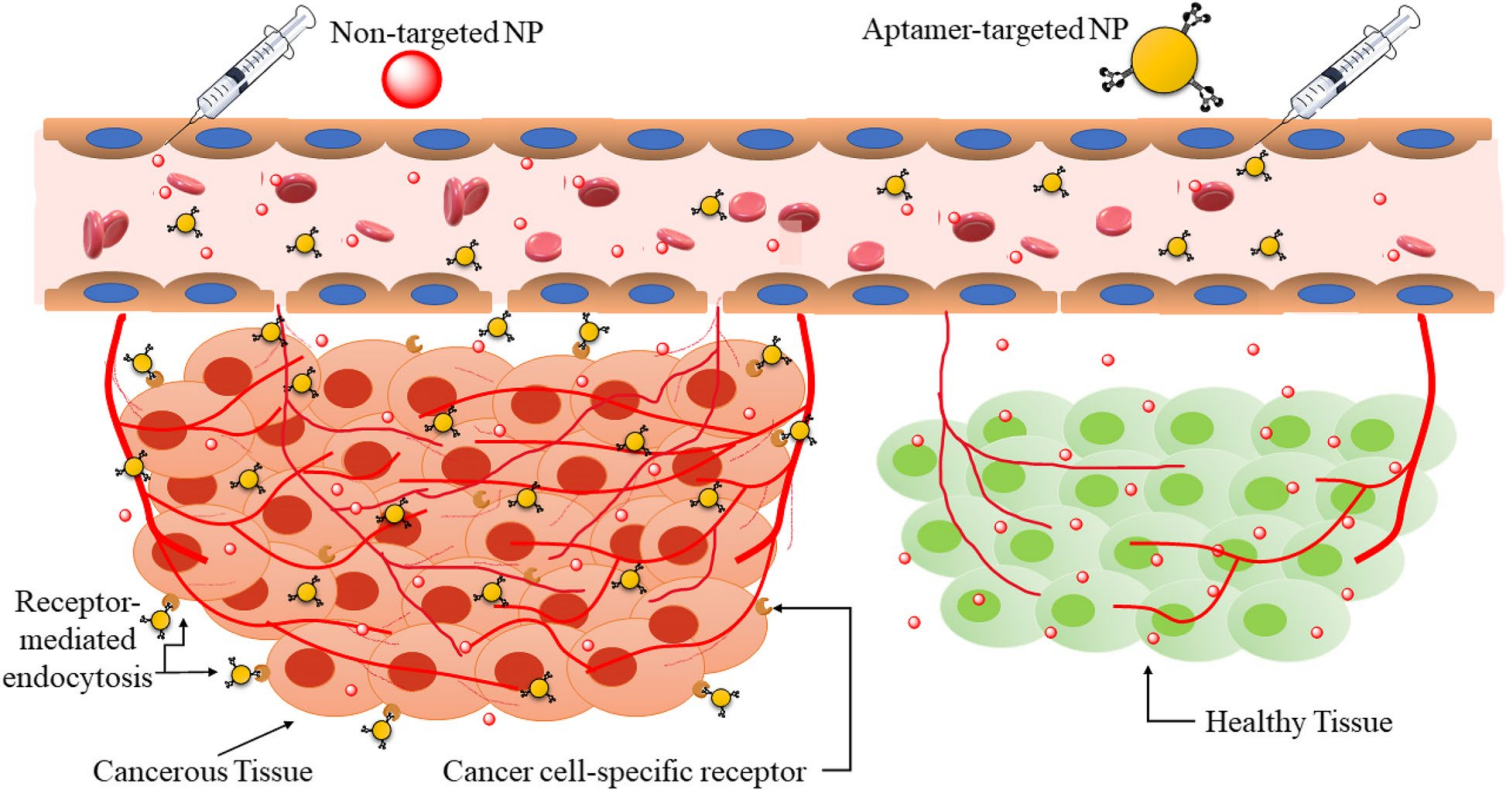

## Introduction

Due to recent rapid development in numerous aspects, frequent advances have been made in treating diseases, including cancer. The high death rate of malignant cancers continues to put patients' lives and health at peril worldwide due to persistent barriers in clinical care and diagnosis [1, 2]. Conventionally used therapies have failed to exhibit success in curing cancer, including therapeutic resistance, metastasis, and recurrence. Looking back in time, cancer evolution has changed our perspectives of the disease while also providing us with a multitude of prospective treatment choices [3]. Cancer treatment began in the early 1900s with the use of radiation therapy, but it took Hertz and Li ~ fifty years to achieve the breakthrough in the complete cure of tumor in 1953. Chemotherapy, a discovery in cancer research used in clinics today, was credited with its effectiveness [4]. Even though these effective treatments and developments in early detection, overcoming the lethal condition remains a formidable challenge. As this therapeutic strategy is non-selective, it harms malignant tissues as well as normal tissues, resulting in non-specific damage. Furthermore, long-term usage of chemotherapeutic medicines might lead to drug resistance in individuals, reducing therapy effectiveness. Systemic and local radiotherapy also gives rise to severe adverse reactions, for example, systemic reactions, radiation osteonecrosis, and radiation pneumonia [5, 6]. Scientists' ability to transfer promising molecular discoveries into advantages for cancer patients is still limited. A treatment paradigm incorporating early clinical diagnosis and suitable therapeutic regimens would enhance prognosis [7]. As a result, it is critical to investigate effective ways for early malignancy diagnosis and treatment approaches to reduce cancer mortality drastically. This might be accomplished using a platform that delivers medicines to cancer cells selectively. Cancer nanomedicine is a versatile instrument that has transformed treatment tactics by escalating its horizons from diagnosis and molecular imaging to drug delivery. It is one of the most actively explored fields of nanotechnology [8]. There are multiple earlier reviews discussing the use of aptamer in diagnostics and theranostics, however, these reviews are limited to particular cancer type or application and do not highlight overall improvement in the efficacy of nanocarrier function due to enhancement with an aptamer [9-12]. The highlight of this review is that it is structured to provide insight into nanoparticle construction and aptamer usage in the context of cancer. This review is unique, in the sense that it provides an elaborate account of aptamer modified nanocarriers (organic and inorganic) and their increased efficacy in cancer targeting by aptamer conjugation.

## Effectiveness of nanoparticle therapeutics

Nanoparticles are extremely minute entities, synthesized chemically, and are composed of miniscule quantities of selective elements that can be measured in nanometer range. Nanoparticles can be imparted with specific properties related to their application. Properties like increased hydrophilicity/hydrophobicity for delivery of compatible drugs to cells or tissues. Nanocarriers can also be loaded with cargo such as drugs, bioactive molecules, siRNA/miRNAs, etc. for site-specific, targeted, and controlled delivery [13]. As nanoparticles can be synthesized chemically, they can be designed to breakdown under specific pH or enzymatic conditions [14]. Because of their ideal size they allow longer circulation time by avoiding renal clearance. Recent breakthroughs in cancer nanotechnology have opened up promising possibilities for precise medication delivery using a new nanotherapeutics domain of passive and active targeting [15, 16]. Improved parameters like solubility, bioavailability, stability, and minimum off-target toxicity are all factors to consider with selective targetability to the desired site and neoplastic cells [17-19]. Nanotherapeutics offer a significant advantage over conventional chemotherapeutic agents [20]. Passive targeting enables the deposition of nanovectors into the tumor surroundings due to unique properties of the tumor microenvironment that are not seen in healthy tissues. The nanocarriers can aggregate preferentially in malignant tissues via passive targeting due to the enhanced permeability and retention (EPR) phenomenon [21, 22]. The EPR is based on the following essential properties of neoplastic tissues: leaky vasculature and poor lymphatic drainage, and the nanometer size range of nanocarriers. The active binding to target cells and uptake by the RES, on the other hand, are two kinetically competing processes. However, receptors or epitopes on the cell exterior allow nanocarriers to identify and attach to target cells via ligand-receptor interactions. To circumvent the limitations of passive targeting, affinity ligands (e.g. small molecules, peptides, antibodies, and aptamers) that selectively bind to particular receptors can be added to the exterior of nanocarriers via various conjugation chemistries [23, 24]. These targeting moieties bind to a biomacromolecule precisely and irreversibly, forming a bigger complex and altering its activity or function [25]. Since small-molecule ligands have a high capacity for developing linkages between proteins, a single ligand can bind to various targets. These molecules not only participate in many essential enzymatic activities (as coenzymes or substrates) to help form metabolic networks but also operate as extracellular and intracellular signals to aid in the construction of regulatory networks [26]. Large molecule ligands, such as proteins, peptides, aptamers, and antibodies, are also used as effective targeting moieties owing to their efficient delivery action to cancer treatment [27]. These cutting-edge gears may be able to create objects with exclusive physical, chemical, or biological properties and tools, allowing for the advancement of innovative diagnoses, treatments, and preventions [28].

### Aptamers: composition, structure, and properties

Aptamers are a novel class of oligonucleotide ligands discovered through rigorous screening and identified for specific cancer cell detection and used with nanotechnological devices at the molecular level as chemical antibodies. These are single-stranded DNA or RNA molecules with a short length (20–60 nucleotide) created utilizing SELEX technologies that bind to various receptors with high affinity and specificity [29, 30]. Aptamers exhibit multipurpose outlays as they have numerous known secondary motifs (loop, stem, or G-quadruplex) that allow them to assume complex 3D configurations and impart high affinity and specificity for target recognition and binding [31, 32]. Aptamers also exhibit similar binding affinities to their cognate targets, such as antibodies. Aptamers offer excellent specificity by distinguishing between enantiomers and proteins with differences of only a few amino acids. Compared to antibodies, aptamers possess low molecular weight, a stable assembly, chemical group flexibility, rapid blood clearance, and non-immunogenicity. Aptamers can increase tumor treatment efficacy and decrease harmful side effects by interacting with tumor cells and associated protein targets [33-35]. They have a wider variety of targets than antibodies. As they do not require animal immunization, they may be made to protect against potent biotoxins. They also need less time to produce since they are made in vitro, eliminating the necessity for extensive animal immunization. Aptamers are not temperature sensitive; they can withstand temperatures up to + 80 °C, whereas monoclonal antibodies will denature at this temperature. Even if denatured, they can readily be renatured to their original three-dimensional assembly. They may be swiftly quirked to improve versatility and stability to exhibit less batch-to-batch variability [36]. Aptamers can be chemically made, and PCR amplified after obtaining the sequence [37, 38].

### SELEX technique/screening of aptamer

**Fig. 1:**
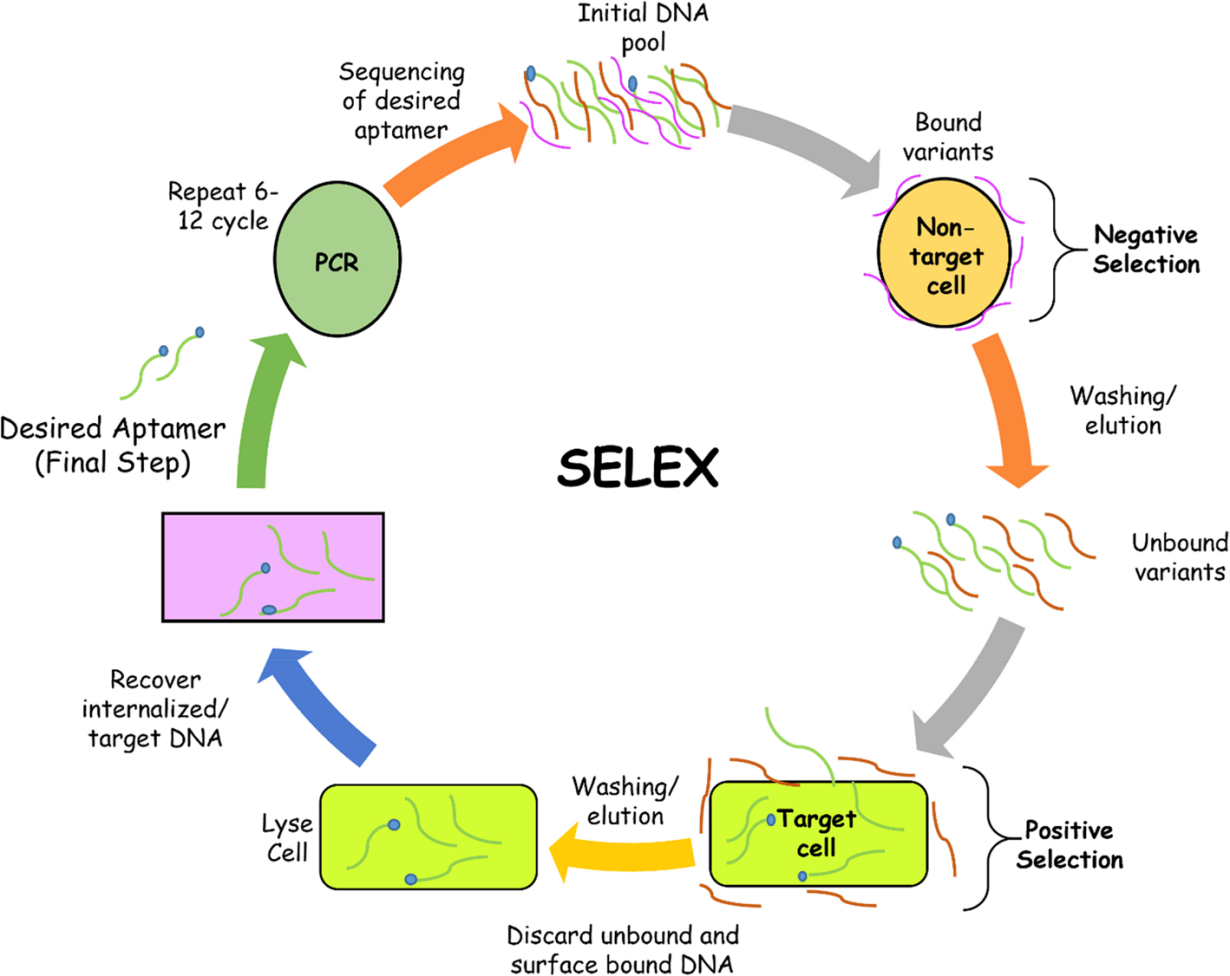
The SELEX method of aptamer selection

SELEX is a general approach for extracting high-affinity single-stranded DNAs or RNAs from a vast library [39]. It is a method for obtaining highly specific aptamers which may be used to select targets such as proteins, cells, viruses, microbes, poisons, and chemical compounds. The method is carried out through a series of positive and negative selection processes. The ideal aptamers for the target are the DNA/RNA oligonucleotides that interact with the target firmly and precisely [40-43]. The SELEX procedure entails (1) target molecules are incubated with a pool of random oligonucleotide sequences (2) discarding of unbound oligonucleotides and the subsequent separation of bound oligonucleotides and (3) PCR amplification of bound oligonucleotides [44-47]. Since the DNA polymerase employed in PCR has a poor fidelity, some alterations are made in individual PCR reactions; consequently, the oligonucleotides binding capability will progressively increase over the selection and amplification process [48, 49]. After their effective selection, their secondary structure, target-binding affinity, stability, and other attributes are examined [50, 51]. The complete SELEX process is represented in Fig. 1.

### Mechanism of action of aptamers 

**Fig. 2:**
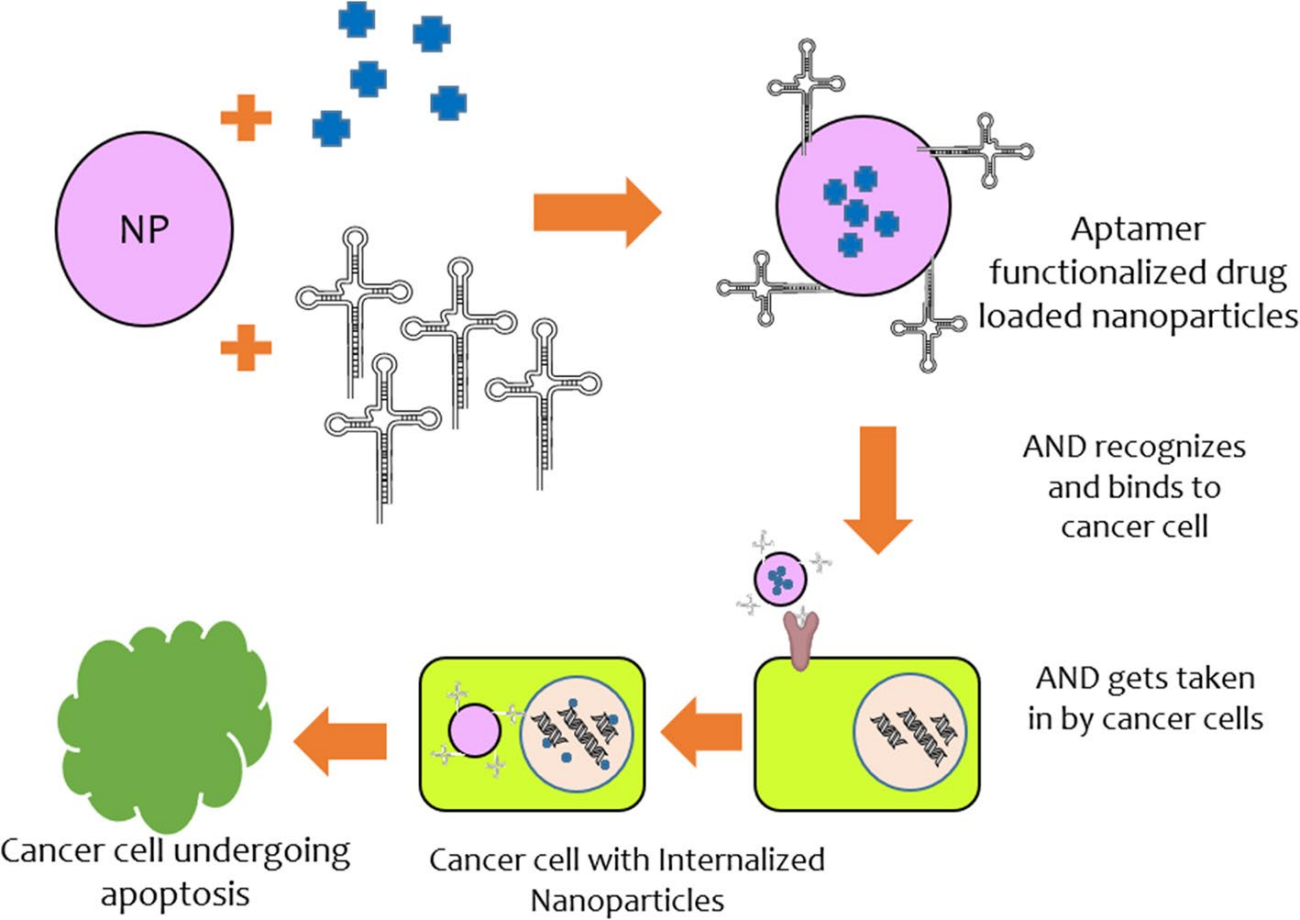
Diagrammatic representation of the cellular absorption of aptamer coupled nanoparticles and cargo delivery inside the cell

Complementary base pairing defines the secondary structure of aptamers, which comprises mostly of short helical arms and single-stranded loops. Because of their stable tertiary structure, which is generated by mixing multiple secondary structures, aptamers bind to the target molecules through van der Waals, hydrogen bonding, and electrostatic interactions. Aptamer-based strategies are typically delivered in one of three ways: (1) An aptamer can be utilized to activate or impede a specific target, such as cancer-related receptors and growth factors. They can also be utilized to inhibit disease-related targets (such as receptor-ligand interactions) from interacting. Aptamers also recognize a variety of targets, including ions, medicines, poisons, peptides, proteins, viruses, bacteria, foreign cells and tissues. (2) Aptamers and medications can be coupled covalently or non-covalently to generate aptamer-drug conjugates (ApDCs). (3) Aptamers can also be used as carriers for therapeutic compounds to be delivered to cancer cells and improve the therapeutic response of new nanoparticles (Fig. 2). Aptamers regularly block protein–protein interactions in binding proteins, producing antagonism effects. A cell-type-specific aptamer can operate as a carrier for delivering other therapeutic compounds to target cells or tissues. Aptamers can operate as drug transporters to epithelial cancer cells by increasing the medication's local concentration in the cells/tissues of interest and stay in the body by interacting with their cellular membrane receptor in a certain way [52, 53]. Many new cancer therapies are focused on delivering therapeutic payloads to and into cancer cells with pinpoint accuracy. The utilization of growth factors, peptide hormones, cytokine, and monoclonal antibodies has supplied a spectrum of ligands needed to target tumor cells specifically. However, the introduction of short synthesized oligonucleotide aptamer ligands has made it easier to find membrane-impermeant aptamers that are specifically designed to target internalized surface indicators generally present on cancer cells. The DNA aptamers, for example, attach to internalized tumor markers such as CD33, CEA, MUC1, and Tn antigens and are introduced into cancer cells via these surface gateways [54, 55].

## Aptamer-modified nanoconstructs in cancer therapy

Conventional anti-tumor drugs are non-specific, and numerous clinical trial studies have revealed considerable harmful effects in their usage. On the other hand, aptamers have a high affinity and selectivity for their target. They have been screened using biomarkers associated with the progression of cancer and can be employed as medicines or coupled with pharmaceuticals, siRNAs, nanoparticles, and other molecules to build a tailored drug delivery system that can target particular tumor cells [56, 57]. As a result, normal cell toxicity is minimized, treatment doses are reduced, and therapeutic efficacy is improved. SELEX has tested a number of aptamers targeting tumor cells, for example, A10 aptamer (against prostate-specific membrane antigen; PSMA), AS1411 aptamer (against nucleolin) [58], EpCAM aptamer (against epithelial cell adhesion molecule), and Sgc8 aptamer (against protein tyrosine kinase 7; PTK7). A number of drug delivery methods have also been created using these aptamers for targeted therapy of precise tumor cells [59, 60]. Aptasensors are a class of biosensors that employs aptamers as bio-receptors and have received a lot of interest in recent years for cancer biomarker detection [61, 62]. Aptamers, like antibodies, are highly precise for binding to targets, as well as have apparent benefits in chemical alteration, stability, and cost of manufacture [63]. Aptamers are being extensively employed in various fields of tumor diagnosis, including the diagnosis of circulating tumor cells (CTCs), immunohistochemical analysis (IHC) of tumor tissues, and in vivo imaging [64].

**Fig. 3:**
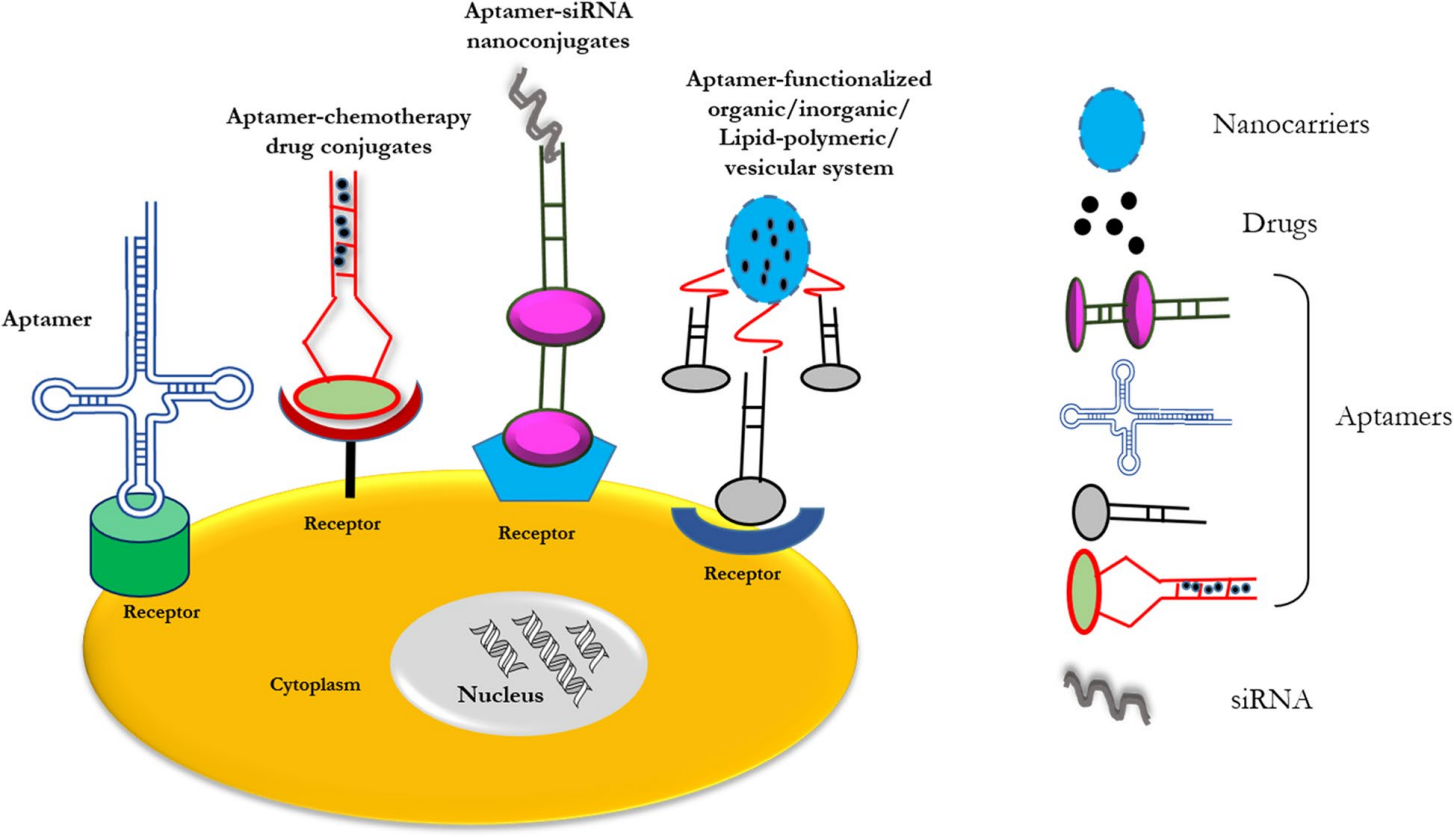
Receptor-mediated endocytosis through the different configured structures of aptamers for the delivery of drugs

Aptamers can be utilized as agonists or antagonists for inhibiting or stimulating the interactions of tumor-associated targets. This function can be utilized by tethering aptamers with drugs to synthesize aptamer-drug conjugates (Fig. 3). Aptamer-tethered nanocarriers having anti-tumor agents, create the tumoricidal effect by leading the therapeutic reagents to the extracellular regions of tumor-specific surface biomarkers. Therapeutic aptamers include aptamer-drug nanoconjugates, aptamer-modified nanocarriers, and aptamer-mediated immunotherapy [65, 66]. Various aptamer-conjugated nanocarriers for anti-cancer drug delivery are discussed in this section.

For the ease of understanding, the review discusses aptamer conjugated nanoparticles by classifying them as organic and inorganic nanocarriers. Organic nanoparticles contain carbon as the central component, whereas, inorganic nanoparticles contain elements other than carbon such as gold, iron, silver, silicon (quantum dots), etc. The group of organic nanoparticles contains polymeric nanocarriers, lipid based nanocarriers and lipid-polymer hybrid nanocarriers. Similarly, the group of inorganic nanoparticles contains metallic nanocarriers, non-metallic nanocarriers, metalloid nanostructures, and molecules directly conjugated with aptamers such as bioactives and drugs. Some nanoparticles are synthesized as a combination of organic and inorganic precursors and are described in related section depending on their functional contributors. Organic and inorganic, both have specific features that can be implemented such as the hydrophobic nature of liposomes and electron dense nature of metal nanoparticles like gold and iron, used in MRI and PET imaging.

### Aptamer-conjugated organic nanocarriers

Organic vesicular nanocarriers, such as liposomes, micelles, lipid-polymer-based carriers, and dendrimers, allow simultaneous treatment and diagnosis (theranostics). Each system has its own characteristics, such as the capacity to transport a wide range of pharmaceuticals and conjugates to targeting agents, as well as being a quick carrier to deliver drugs for cancer therapy with little damage. These nanoformulations effectively incorporate chemotherapeutic drugs, small interfering RNAs, biologicals, imaging molecules, and specific NPs, such as superparamagnetic NPs. These nanocarrier devices are effective not just for passive targeting but also for active targeting. Herein, the research and tactics in aptamer-modified polymer/lipid‐based nanocarriers for cancer treatment are discussed.

#### Polymeric Nanoparticles

Dendrimers are distinct synthetic macromolecules posing a repetitive molecular configuration and huge surface functional groups. Over the last decade, dendrimers have proven to be an excellent carrier for various bioactives. Drug entrapment and release from the dendrimer may be modulated by changing the surfaces and generations of dendrimers. For designing and engineering an ideal nanodevice, targeting ligands and imaging molecules can be attached readily to dendrimers. Dendrimers offer the potential to produce next-generation nanodevices due to their multifunctional capabilities [67].

There are several exciting studies on aptamer attached dendrimers for cancer treatment. Few of them are discussed in this section. Clinical application of various cancer drugs (e.g., epirubicin) has been restricted due to complications like cardiotoxicity. In a novel approach, Taghdisi et al. engineered an upgraded DNA dendrimer with three different aptamers, namely, MUC1, AS1411, and ATP aptamers [68]. The dendrimers were designed for targeted drug delivery of epirubicin to MCF-7 (breast cancer) and C26 (murine colon cancer) cells. The DNA dendrimers were synthesized step-wise up to G3 and loaded with epirubicin (25 µM) at different concentrations. The drug-loaded dendrimers were reacted with the aptamers, and the Apts-Dendrimer-epirubicin complex was assessed by electrophoresis. The drug release study revealed that 63% and 15% epirubicin was released at pH 5.5 and 7.4, respectively. The cell viability of MCF-7 after treatment with Apts-Dendrimer-epirubicin complex showed higher specificity towards MCF-7 and C26 cells with 65–70% toxicity. Furthermore, the targeted conjugate showed high biocompatibility of > 85% with CHO cells. The most crucial finding of this study was the in vivo assessment of single aptamer tagged dendrimer and doubly tagged dendrimer. The MUC1 aptamer-Dendrimer-epirubicin conjugate and AS1411 aptamer-Dendrimer-epirubicin showed 1802mm^3^ and 850mm^3^ of tumor volume after 16 days of treatment. On the other hand, the dendrimer tagged with both aptamers showed a reduced tumor volume of 585mm^3^. Thus, the work done by this group provides strong evidence of the increased effectivity and specificity of drug-loaded dendrimers upon their modification with single and double aptamers.

Zhang et al. have engineered a multifunctional DNA dendritic nanostructure for targeted cancer cell imaging and drug delivery [69]. Herein, unique Y-shaped DNA monomers were hybridized repetitively with other Y-DNA segments up to G3 to form DNA dendrimers. The aptamers (sgc8) were added to the solution to create a self-assembled aptamer-based nanostructure. These DNA dendrimers showed a single band on the agarose gels implying high purity and reaction efficiency. The average size of G1, G2, G3, and G3-sgc8 dendrimers was 13.7, 21.0, 24.3, and 43.8 nm, respectively. The aptamer-modified dendrimer showed ~ 40 × fluorescence in the cancer cell line; in contrast, no fluorescence was seen in Ramos cells either with free sgc8 and G3-sgc8. Thus, with facile design and preparation, the DNA-dendrimer shows high specificity and multifunctionality.

**Fig. 4:**
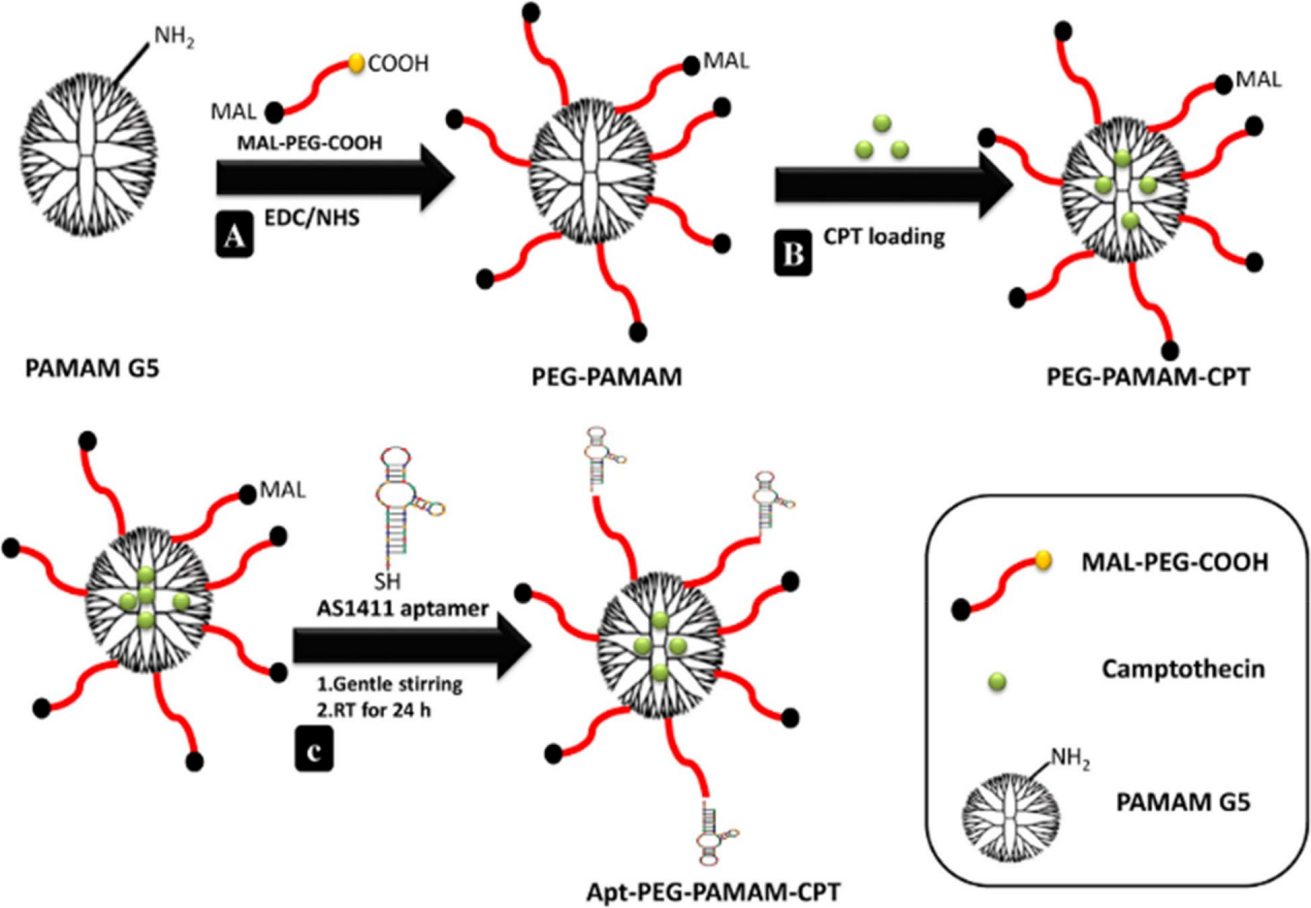
Schematic representation of (**A**) synthesis of pegylated PAMAM dendrimer (PEG-PAMAM); (**B**) camptothecin (CPT) loading in the cavities of PEG-PAMAM; (**C**) conjugation of thiolated AS1411 aptamers to the maleimide groups of MAL-PEG-PAMAM-CPT and preparation of Apt-PEG-PAMAM-CPT. Reproduced with permission from Alibolandi et al. [70]. Copyright Elsevier (2017)

In the study by Alibolandi et al., camptothecin-laden PEGylated PAMAM dendrimers (Fig. 4) were surface modified with AS1411 (anti-nucleolin aptamers) to target colon adenocarcinoma [70]. The size was 14.2 and 18 nm for PEG-PAMAM-CPT and Apt-PEG-PAMAM-CPT, respectively. The PEGylated dendrimers showed increased camptothecin loading compared to plain dendrimeric structure, with a drug loading of 8.3% and entrapment efficacy of 98.7%. Both produced nanoformulations, PEG-PAMAM-CPT and Apt-PEG PAMAM-CPT, demonstrated regulated CPT release over 4 days. In PBS pH 7.4, PEG-PAMAM-CPT released 95.48 percent of the drug during 4 days, whereas Apt-PEG PAMAM-CPT released 73.15% of the drug during 4 days. In cellular uptake studies, the fluorescent intensities of Apt-PEG-PAMAM-Dil treated HT29, and C26 cells were 73% and 81%, respectively. The fluorescent intensities of non-targeted PEG-PAMAM-Dil treated HT29 and C26 were 52% and 64%. Thus, the aptamer conjugated nanoconstructs showed higher uptake. The IC_50_ value in the HT29 cell line measured through the MTT assay was 15 µg/ml, 10 µg/ml, and 3 µg/ml for free CPT, PEG-PAMAM-CPT, and Apt-PEG-PAMAM-CPT, respectively. For C26 cells, the IC_50_ value was 18 µg/ml, 8 µg/ml, and 1.5 µg/ml for free CPT, PEG-PAMAM-CPT, and Apt-PEG-PAMAM-CPT, respectively. Thus, these novel nanoconstructs showed high versatility and increased specificity using AS1411 ssDNA aptamer. Similarly, Mohammadzadeh et al. conjugated the ALGDG2 dendrimer with AS1411 aptamer for targeting Iohexol for imaging [71]. Iohexol or omnipaque is an iodinated contrast reagent that aids in diagnosis through a CT scan. The multifunctional nanocarrier tackles the drawbacks of imaging agents' secluded use, such as rapid clearance, toxicity, and non-specificity. The in vitro cytotoxicity was assessed through the XTT assay for 24, 48, and 72 h. While there was no cytotoxicity after 24 h, significant toxicity was observed at higher concentrations after 48 and 72 h on both the cell lines (MCF-7 and HEK-293). However, Apt-ALGDG2-Iohexol showed no cytotoxicity to the normal HEK-293 cells after 72 h showing high biocompatibility towards normal cells. The non-targeted nanoconstructs showed toxicity to HEK-293 cells at 100 µM and 20 µM concentrations. Remarkably, increased survival of ~ 92% was observed in normal cells for the Apt-ALGDG2-Iohexol by the Annexin V-FITC labeling in flow cytometry. In contrast, HEK-293 cells treated with free Iohexol had undergone late-apoptosis/necrosis up to ~ 90%. The targeted Apt-ALGDG2-Iohexol did not show any in vivo cytotoxicity when injected into the 4T1 breast tumor model. The images showed a high accumulation of the targeted NPs in the tumor and bladder, confirming high specificity and rapid clearance from the body. Thus, the dendrimers could be a cheap, effective, and efficient nanoconstruct tool for implementing aptamers in nanotheranostics.

Miyano et al. developed a negatively charged sixth-generation amino acid dendrimer- KG6E, based on glutamic acid-modified dendritic poly(L-lysine) system [72]. Further modification of KG6E was done by conjugating an anti-mucin 1 (MUC1) aptamer by Masuda et al. in a separate study for the targeted drug delivery to lung cancer A549 cells which express high levels of MUC1 [73]. The aptamer was extended by 20 adenine nucleotides to extrude it and ensure access for efficient binding to MUC1. Flow cytometry data showed significant specificity of the aptamer tagged dendrimer towards the A549 cells. As visualized from confocal microscopy, the A549 cells efficiently internalized the anti-MUC1 apt/KG6E, which was then delivered to the endo-lysosomal compartment of the cell. However, the association of the scrambled aptamer/KG6E nanoconjugates to cells was significantly inferior to that of the anti-MUC1 apt/KG6E. The findings suggested that the anti-MUC1 apt/KG6E is internalized readily in A549 cells through a MUC1 recognition that can aid in targeted cargo delivery and detection of cancer tissues. Behrooz et al. constructed a smart carrier of AS1411 aptamer conjugated 5FU-loaded PAMAM dendrimers for stomach cancer therapy [74]. The aptamer conjugated dendrimers had a mean particle size of 2965 nm, a zeta potential of -13.6 mV, and a PDI of 0.76. The EE of 5FU was found to be 76.8%. The cellular absorption of aptamer coupled dendrimers by MKN45 cells rose by 98.78% compared to the control group. MKN45 cells were 77%, 82%, and 81.5% cytotoxic to free 5FU, 5FU dendrimers, and aptamer attached 5FU dendrimers. The cytotoxicity was decreased following aptamer conjugation. However, in vivo drug deposition was much more significant after 60 min when Apt conjugated dendrimers were utilized to treat MKN45 tumors than non-conjugated dendrimers.

In a venture to synthesize a stable multifunctional nanomaterial, Zhou et al. developed an aptamer-dendrimer conjugate capable of carrying multiple signal molecules [75]. These conjugates have a size of ~ 8 nm and bind to target cancer cells efficiently, helping in their detection and diagnosis. The group reported specific binding of the hybrid nanomaterial to CCRF-CEM and Ramos cells. Similarly, Li et al. used the quantum dot (QD) modified dendrimers and conjugated them with aptamers for imaging glioblastoma cells [76]. The group conjugated the QD modified dendrimer with GBI-10 aptamer that specifically binds to tenascin-C, an extracellular protein that plays a crucial role in cell proliferation and migration. The dendrimers were modified with quantum dots to negate their cytotoxicity and increase their cellular uptake. The nanoconjugates could successfully bind to cancer cells in vitro. These studies suggested the limitless potential in the applicability of dendrimer-aptamer complexes.

Zhang et al. used a PEI-polymer to create a nanocomplex comprising ATP sensitive aptamer and its cDNA, DOX, and Bcl-2 siRNA to accomplish targeted drug delivery for drug-resistant prostate cancer treatment [77]. The developed nanocomplex having a zeta potential of + 9.85 mV and an average particle size of 360.8 nm. The effect of ATP on DOX release from nanocomplexes was examined, and it was shown that increased ATP concentrations resulted in more drug release in the cytoplasm of cancer cells. The cytotoxicity of the nanocomplex was tested using PC-3 cells. Post-treatment with the DOX-duplex and polymer-siRNA complex, the viable cell count was found at 98.9% and 77.5%, respectively. After treatment with polymer coupled DOX-Duplex, the viable cell count was 62.5%. Cell viability was 53.3% after treatment with the DOX-Duplex-siRNA polymer nanocomplex at 5 µg/mL DOX and 15 µg/mL siRNA doses. The DOX-Duplex-siRNA polymer nanocomplex has a synergistic effect, according to these findings. Bcl-2 expression was reduced in PC-3 cancer cells after treatment with polymer siRNA complex and DOX-Duplex-siRNA polymer nanocomplex. Apoptosis rates in PC-3 cells were measured by flow cytometry, and were found to be 3.97%, 7.88%, 20.15%, and 36.23% in the control, polymer siRNA nanocomplex, polymer DOX-Duplex complex, and DOX-Duplex-siRNA polymer nanocomplex groups, respectively. The significant percentage of apoptosis observed after treatment with the DOX-Duplex-siRNA polymer nanocomplex corroborated DOX-Duplex and siRNA's synergistic effect. The DOX-Duplex-siRNA polymer nanocomplex enhanced the G2 ratio to 37.67% and induced cell cycle arrest in the G2 phase in the cell cycle arrest assay, whereas the polymer siRNA nanocomplex had no such effect. The results demonstrated that aptamer ATP responsiveness and cDNA-DOX conjugation may be used to induce a synergistic anticancer impact in PC-3 cells.

Subramanian et al. produced EpCAM siRNA-loaded PEI polymeric NPs conjugated with aptamer [78]. The NPs were spherical with a hydrodynamic diameter of 151 ± 11 nm. Their results suggested that MCF-7 cells absorbed siRNA aptamer-NPs much more quickly than WERI-Rb1 cells. Further, MCF-7 cells treated with EpCAM-aptamer conjugated EpCAM siRNA-NPs had a 64% gene downregulation, compared to 56% in cells treated with EpCAM siRNA. Similarly, gene downregulation findings in WERI-Rb1 cells demonstrated high effectiveness with EpCAM-aptamer coupled siRNA-NPs (72%) as compared to EpCAM siRNA-NPs (62%) in cells. The EpCAM-aptamer coupled siRNA-NPs successfully targeted EpCAM tumor cells, delivered siRNA, silenced the target gene, and repressed cell proliferation more than the scrambled aptamer loaded nano-construct, signifying its efficient targeting ability to EpCAM receptor.

**Fig. 5:**
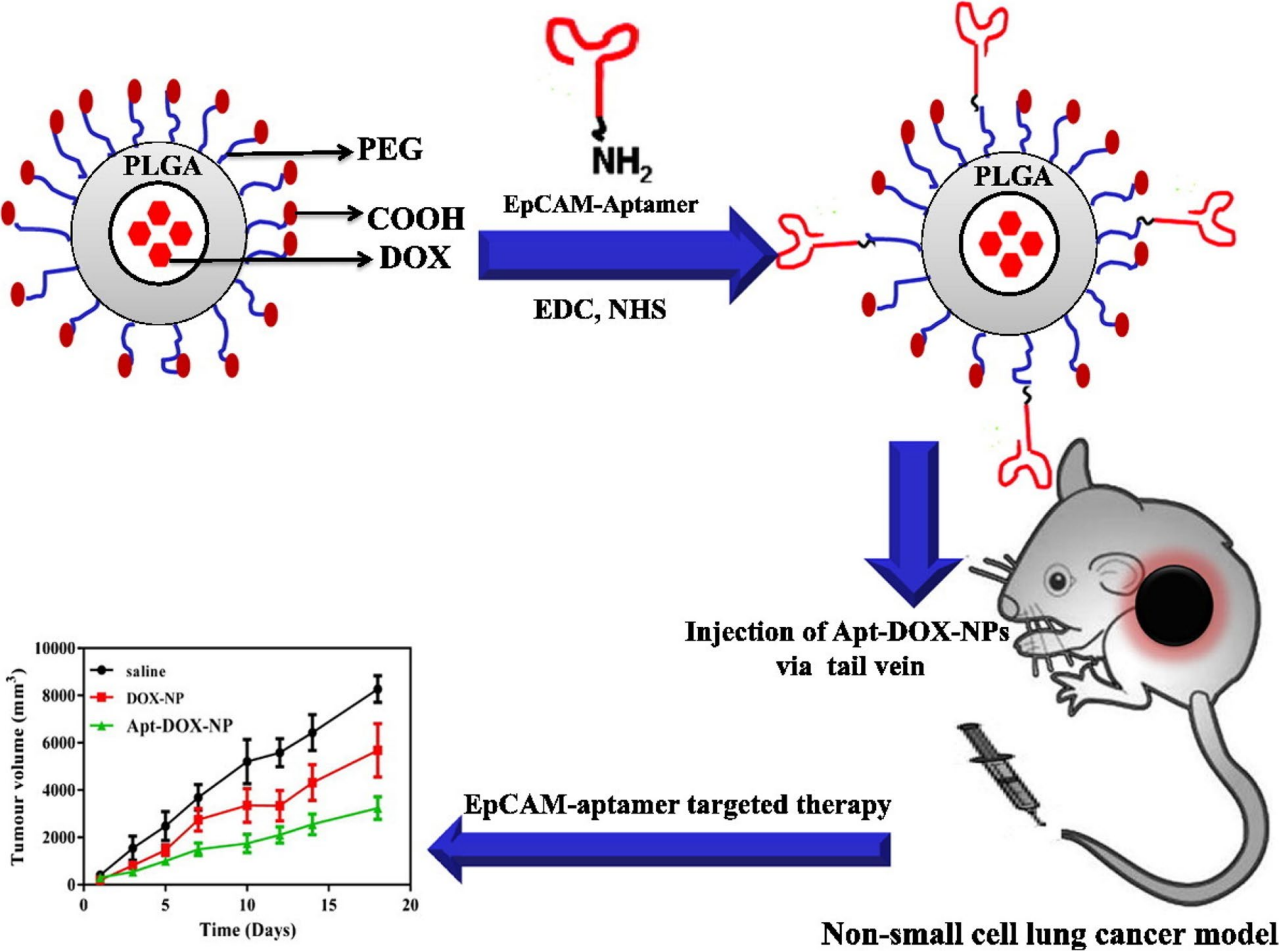
Graphical representation of the work done Alibolandi et al. [79]. Copyright Elsevier (2015)

Alibolandi et al. used a thin film hydration technique to create EpCAM-Apt targeted DOX loaded nanocarriers for the treatment of non-small cell lung cancer (NSCLC) (Fig. 5) [79]. The synthesized EpCAM RNA apt conjugated PLGA-*b*-PEG nanopolymerosomes had a mean particle size of 136 ± 0.21 nm, a PDI of 0.12, and zeta potential of 36.13 mV. The EE and DL of DOX were obtained to be 91.25 ± 4.27% and 7.3 ± 0.34%, respectively. DOX release from NPs was faster at pH 5.5 when related to pH 7.5. After 5 days, around 15% of the medicine had been released, indicating a biphasic release pattern. Flow cytometry evaluated specific EpCAM-Apt binding to EpCAM expressing SK-MES-1 and A549 small lung cancer cells. In SK-MES-1 cells, EpCAM-Apt binding was found to be 46.12%, whereas in A549 cells, it was obtained to be 54.27%. In an in vitro cellular uptake study, Apt-NPs increased cellular absorption by 83% in SK-MES-1 cells and 78% in A549 cells compared to a negative control group. In contrast, cellular absorption of non-conjugated NPs increased by 52% in SK-MES-1 cells and 46% in A549 cells. In vitro cytotoxicity of Apt-NPs against both SK-MES-1 and A549 cell lines was much greater than that of non-conjugated NPs. The in vivo anticancer impact of NPs was investigated using SK-MES-1 cells in C57BL/6 nude mice. The Apt-DOX-NPs group had a tumor volume of 2000mm^3^, whereas the DOX-NPs and normal saline groups had tumor volumes of 5000mm^3^ and 8000mm^3^, respectively. The Kaplan–Meier survival curves showed that the group treated with Apt-Dox-NPs had a 100% survival rate until 21 days, then dropped to 50% after 30 days. The group given Dox NPs had a 100% survival rate for the first 15 days, then dropped to 25% after 30 days. The survival rate of the group given free Dox was 70% on the first day, 40% in 11 days, and all rats died within 21 days. The results clearly demonstrated the superiority of Apt-DOX-NPs formulations over DOX NPs and free DOX alone.

Raţa et al. used an interfacial condensation technique to make AS1411 aptamer-functionalized polymeric nanocapsules (NC) of 5-flurouracil (5-FU) for tumor treatment [80]. The mean diameter of produced NC was obtained to be 133 ± 3.46 nm with a PDI of 0.33 ± 0.020 and zeta potential of 19.2 ± 0.12 mV. The EE and DL of 5-FU were obtained at 22.5 ± 0.86% and 0.34 ± 0.01, respectively. In PBS (pH 7.4), the prepared NC formulation demonstrated burst release (15%) of 5-FU followed by continuous release (25%) in 24 h. After 5 h of incubation at a 500 µg NC/mL concentration, the hemolysis produced by NC was only 0.49%, indicating that the prepared NCs were safe to use. The in vitro cytotoxicity of NC was investigated on human dermal fibroblasts. The cells treated with NCs at a 500 g NC/mL concentration exhibited 75% viability, which indicated that NCs were non-toxic in nature. After 48 h, neoplastic MCF-7 cells treated with NCs at a 100 µg/mL dose were only 57.74% viable, whereas free 5-FU showed only 52.88% viability.

Das et al. created aptamer conjugated polymeric NPs for cancer theranostic approach, better cancer treatment, and targeted drug delivery [81]. They used a single emulsion solvent evaporation technique to produce polymeric (PLGA) NPs of nutlin-3a. The EpCAM aptamer (sequence 5′-amino-C12-GCG ACU GGU UAC CCG GUC G-idT-3′) and quantum dots (QDs) were then coupled on the surface of the PLGA-NPs. The prepared nutlin-3a aptamer-NPs (Apt-Nut-NPs) had a mean particle size of 292 ± 10 nm, a PDI of 0.36 ± 0.02, a zeta potential of 20.3 ± 0.66 mV, and a drug EE of 51.24 ± 6.7%. In vitro drug release from Apt-Nut-NPs was examined. The result indicated a burst release of 20% in an hour followed by a steady release of 55% in 72 h with 10% FBS. Apt-Nut-NPs were tested for cellular absorption in ZR751, MCF-7, SKOV3, and HEK-293 cell lines in vitro. Apt-Nut-NPs were considerably more readily taken up by EpCAM positive ZR751, MCF-7, and SKOV3 cells than non-conjugated NPs. Meanwhile, EpCAM negative HEK-293 cells, showed no significant difference in cellular absorption of Apt-NPs and non-conjugated NPs. In ZR751, MCF-7, and SKOV3 cells, the IC_50_ value of Apt-Nut-NPs was reduced by 4 × , 3 × , and 2 × , respectively, in comparison with non-conjugated Nut-NPs. Apt-Nut-NPs induced apoptosis at a rate of 21.08%, whereas Nut-NPs induced apoptosis at a rate of 14.5%. The increase in BAX overexpression, caspase 3 cleavage, and bcl2 downregulation following treatment with Apt-Nut-NPs was verified by western blotting methods as linked to cells treated with Nut-NPs.

Engelberg et al. developed an aptamer decorated block-copolymer PEG-PCL NPs loaded with paclitaxel (PTX) via nanoprecipitation technique for targeted medication administration to NSCLC patients [82]. They conjugated Cy5 tagged S15-Aptamers on NPs for Clathrin-mediated endocytosis. The synthesized NPs had a mean particle size of 30 ± 1 nm and zeta potential of 29.2 ± 0.6 mV. The EE and DL were obtained to be 77 ± 13% and 35 ± 6 (µg PTX/mg PEG-PCL), respectively. A comparative cellular uptake investigation was conducted for the cellular uptake study, and it was discovered that cellular absorption of S15-APTs attached NPs was significantly greater in A549 cells than BEAS2B, FSE, and HEK-293 cells, as well as malignant HeLa and CaCo-2 cells, demonstrated that S15-APTs was an aptamer unique to A549 cells. The IC_50_ values of aptamer conjugated PTX NPs against A549, BEAS2B, HeLa, CaCo2, FSE, and HEK-293 cells were obtained at 0.03, 1.7, 4.2, 43, 87, and 980 µM of PTX, respectively. These findings showed a 2 to 5 order of magnitude difference in NSCLC-specific cytotoxicity, indicating a potentially excellent treatment. Their findings implicated that these APT-decorated NPs have a lot of preclinical potential for selectively targeting and eradicating human NSCLC cells while causing no damage to normal tissues.

Chakraborty et al. studied the capability of the aptamer TLS 9a (L5) conjugated and PTX loaded PLGA-PEG based nanocarriers for therapeutic management of hepatocellular carcinoma (HCC) [83]. PTX-NPsL5 showed a mean size range of 156.9 ± 42.97 nm and zeta potential of 14.5 ± 0.51 mV, respectively. For a period of 60 days, the cumulative percentage of drug released from PTX-NPs and PTX-NPsL5 was determined to be 72.79 and 77.77%, respectively. The PTX-NPsL5 formulation exhibited the highest apoptotic potential when compared to the PTX-NPs formulations, according to the results of the apoptosis investigation. Furthermore, the maximum internalization of NPs by neoplastic hepatocytes and minimal internalization of NPs by normal hepatocytes suggested that it could preferentially target neoplastic hepatocytes. PTX-NPsL5 produced substantial apoptosis in both kinds of neoplastic hepatocytes, with percentages of apoptotic cells of 71.6 (for HepG2 cells) and 73.1 (for Huh-7 cells) percent. The results of in vivo investigations demonstrated that PTX-NPsL5 decreased tumor incidence and progression. In this investigation, the potential of PTX-NPsL5 to establish itself as a targeted treatment to considerably prolong survival in HCC was demonstrated.

#### Lipid based nanocarrier systems

Liposomes are the most widely used vesicular systems. Liposomes protect various drugs against chemical and immunological breakdown, as well as the actions of enzymes. The use of "stealth" liposomes can further reduce toxicity and minimize drug dosage [84, 85]. Liposomes can be employed for selective targeting to cancer cells by attaching ligands (e.g., aptamers, antibodies, peptides, and carbohydrates) to the exterior or the terminal end of the associated PEG chains. This section highlights the recent studies in which aptamer-tethered liposomes were employed for cancer therapy.

**Fig. 6:**
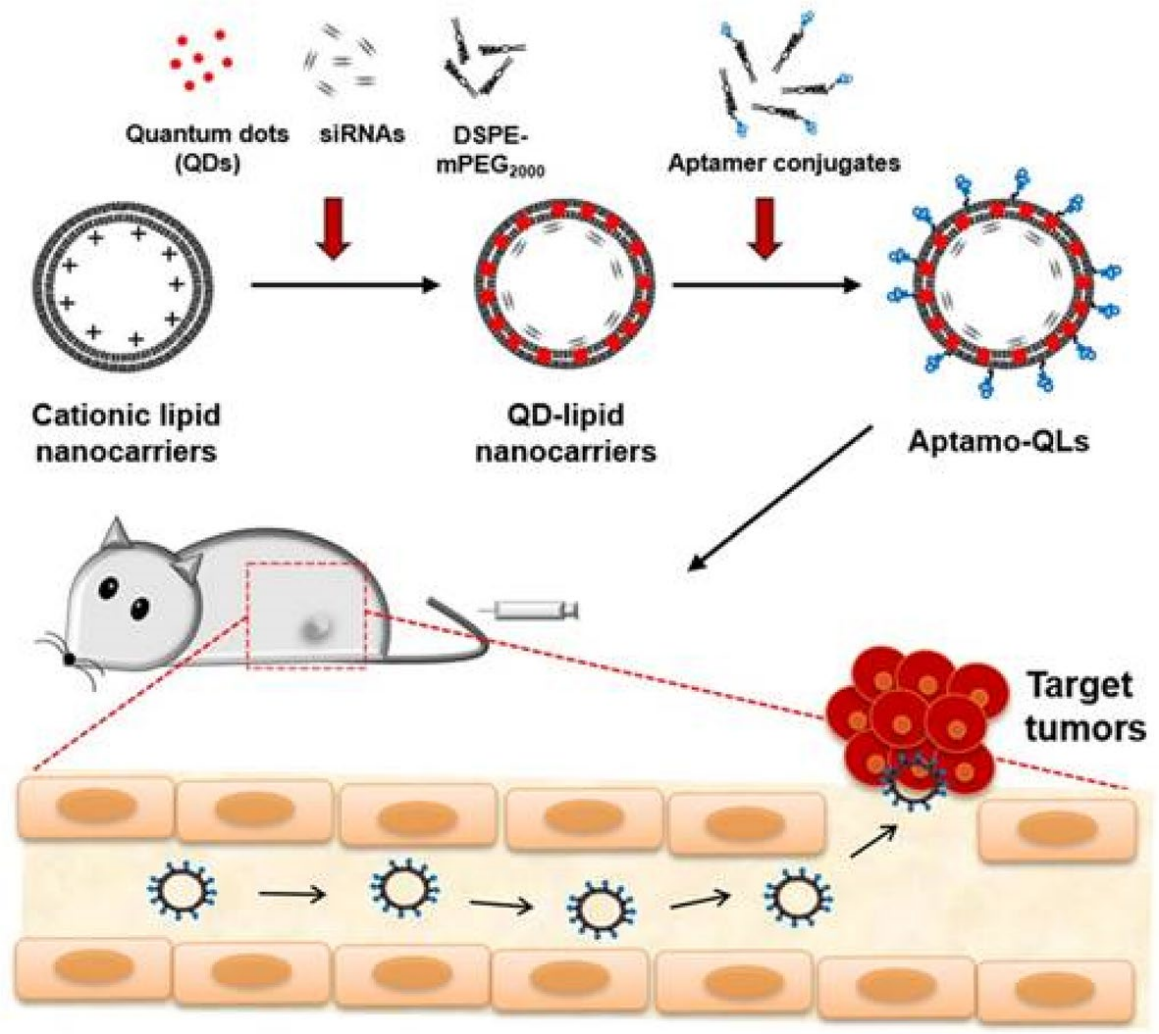
Schematic of the theranostic strategy for RNAi gene therapy and fluorescence tumor imaging. Anti-EGFR aptamer-conjugated lipid nanocarriers encapsulating QDs and anti-cancer siRNAs were prepared and intravenously administered. The administered lipid nanocarriers presumably extravasated through the leaky tumor vasculature and then targeted primary tumors via specific recognition of EGF receptors overexpressed on MDA-MB-231 tumors. The delivered QDs and therapeutic siRNAs provide fluorescence tumor images and inhibitory effects on tumor growth. Reproduced with permission from Kim et al. [88]. The figure is reproduced under Creative Commons Attribution License

Yamada et al. created dual ligand decorated liposomes with a cell piercing peptide and mitochondrial targeting RNA aptamer [86]. They used the thin film hydration technique to make Liposomes. They utilized a mitochondrial delivery device called a MITO-Porter, which has an octaarginine-modified surface (R8), a device that increases cellular uptake. The prepared liposomes were 100–150 nm in diameter with a negative charge on the surface. They also looked at how editing RNA aptamers with R8 modified MITO-Porter improved cellular uptake of liposomes in HeLa cells. Clathrin-mediated endocytosis was liable for the majority of the cellular uptake. The CLSM pictures suggested aptamer coupled liposomes targeting mitochondria. However, when they used carbonyl cyanide 4-(trifluoromethoxy) phenylhydrazone (FCCP), it caused depolarization of the mitochondrial membrane potential, which prevents dual ligand attached liposomes from targeting mitochondria. They also discovered that oligomycin treatment depleted ATP, which reduced the mitochondrial targeting of dual ligand attached liposomes. In the existence of FCCP and oligomycin, cellular absorption of dual ligand attached liposomes was reduced by 20%, indicating that cellular uptake was mostly through endocytosis and macropinocytosis. Naznin et al. created aptamer-conjugated liposomes for tumor targeting via endothelial cells [87]. The endothelial cells of the tumor were targeted using the Ara HH001 aptamer. Thin lipid film hydration was used to make the liposomes. The cellular uptake of aptamer attached liposomes was investigated using a spectrophotometer and a Rhodamine test. When mouse tumor endothelial cells (mTECs) were treated with aptamer coupled liposomes, their fluorescence was 3.8 times greater than those treated with regular liposomes. The aptamer coupled liposomes were found in the lysosomal compartment of mTECs cells and showed increased cellular absorption according to CLSM pictures. In addition, they performed a competitive uptake experiment in mTECs using Rhodamine labeled aptamer attached liposomes. The findings showed that cellular uptake is primarily mediated by endothelial cell receptor-mediated endocytosis. CLSM demonstrated in vivo aptamer targeting capacity to epithelial cells in human renal cell carcinoma harboring mice following IV injection of aptamer attached liposomes. According to the pixel counting, regular liposomes attach to TECs at a rate of 3%, while aptamer coupled liposomes bind at a rate of 25%. Herein, aptamer affinity for TECs was responsible for the increased binding. Kim et al. developed anti-EGF aptamer-guided delivery of siRNA-loaded quantum dots (QDs) encapsulated in liposomes to treat triple-negative breast cancer (TNBC) [88]. They attached aptamer to the surface of liposomes after complexing siRNA to them (Fig. 6). The produced liposomes had an average diameter of 175.5 nm, PDI of 0.37 ± 0.05, and zeta potential of − 1.9 ± 0.7 mV. Liposomes had a QDs integration rate of 94.9 ± 4.8%. The aptamer target-specific delivery was investigated over EGFR-positive MDA-MB-231 and EGFR-negative MDA-MB-453 cells. EGFR-positive MDA-MB-231 cells subjected to aptamer attached liposomes had a mean fluorescence intensity (MFI) of 274.5, whereas EGFR-negative MDA-MB-453 cells treated with aptamer attached liposomes had an MFI of 19.6. This validates aptamer's EGFR selectivity. The CFM images revealed that clathrin-mediated endocytosis was the pathway for cellular uptake. Further, the in vivo fluorescence imaging revealed that aptamer coupled liposomes were capable of successfully targeting malignancies. Similarly, Alshaer et al. also reported siRNA delivery via aptamer-guided liposomes for gene silencing in CD44 expressing murine TNBC model [62]. They used an anti-CD44 aptamer (Apt1) with 2'-F-pyrimidines as a targeting moiety. The mean particle size of prepared liposomes was 137 ± 3 nm with a PDI of 0.085 ± 0.03 and zeta potential of − 26.8 ± 2.3 mV. This non-cationic methodology was tried in vitro and in vivo for silencing the luciferase (luc2) reporter gene in TNBC model. They investigated gene silencing in MDA-MB-231-Luc2-eGFP cells in vitro. Luciferase activity results indicated reduced action to 25.7 ± 15.1% with aptamer coupled siRNA liposomes, and to 47.2 ± 10.6% after treatment with non-conjugated siRNA. Female nu/nu mice were used to study in vivo gene silencing in the MDA-MB-231-Luc2-eGFP-based breast cancer paradigm. After 4 days of treatment with siRNA loaded aptamer coupled liposomes, relative luciferase activity was 150%, whereas it was more than 500% in the group treated with non-conjugated siRNA liposomes. Their result established the possibility of conjugating an aptamer to siRNA-containing liposomes for efficacious gene silencing in CD44-expressing tumor cells in vivo.

Cadinoiu et al*.* used the AS1411-SH aptamer-functionalized liposomes for the targeted delivery of Fluorouracil (5FU) for basal cell carcinoma treatment (BCC) [89]. The liposomes were prepared by the thin-film hydration technique. The mean particle size of optimized batch liposomes was found to be 182 ± 27 nm with a PDI of 0.16 and zeta potential of − 12.9 ± 1.3 mV. Liposomes showed conjugation effectiveness and drug loading (DL) of 37.2 ± 3.7% and 8.3 ± 1.0%, respectively. The quantity of 5FU that entered the model membrane per cm^2^ after 7 days was found to be 60.2 g/cm^2^ in the treatment group L4Apt-5FU-15. The non-toxicity of produced liposomes was demonstrated by the hemolysis generated by aptamer coupled 5FU liposomes at a maximum lipid concentration of 500 µg/mL after 5 h of incubation. Human dermal fibroblast cells (HDFa) were used to test the cytotoxicity of produced liposomes (HDFa). HDFa cells were 97% viable when treated with blank aptamer conjugated liposomes at a lipid concentration of 500 µg/mL. At a lipid concentration of 250 µg/mL, HDFa cells treated with aptamer attached 5FU liposomes were only 15% viable, and 0% after 500 µg/mL lipid concentration treatment. HDFa cells were also tested for apoptosis-induced cell death. The proportion of apoptotic cells in HDFa cells treated with aptamer attached liposomes (in which the lipid concentration was 200 µg/mL and the 5FU concentration was 200 µg/mL) was 15%, but it was found only 8% following treatment with pure 5FU at 500 µg/mL. According to the flow cytometric test, the cellular absorption of aptamer attached liposomes was considerably greater than regular liposomes in HDFa cells. They also tested the antitumor effect of prepared liposomes on TE 354 BCC in vitro. The TE 354 cells were 48% viable after 48 h of incubation with aptamer coupled 5 FU liposomes at 100 µg/mL lipid content and 60% viable after 48 h of treatment with conventional 5FU liposomes. All the results proved the efficient delivery of 5FU via aptamer conjugated formulation.

Liang et al. reported an aptamer governed nanostructured lipid carriers (NLCs) of epigallocatechin gallate (EGCG) by emulsion solvent evaporation method followed by sonication [90]. They used control DNA of ATP aptamer (ACC TTC CTC CGC AAT ACT CCC CCA GGT) and also HER2 receptor targeting HB5 aptamer (AAA AAA AAA AAA AAA CGT GCA GTA CGC CAA CCT TTC TCA TGC GCT GCC CCT CTT AAG TAC GCC AAC CTT TCT CAT GCG CTG CCC CT). The prepared NLCs had a hydrodynamic diameter of 190.44 nm and a zeta potential of -9.21 mV. The two aptamers specific to HER2 and ATP were assembled in a hierarchical way in this nanovehicle. The identification of HER2 protein overexpressed in SK-BR-3 cell lines is governed by the outmost HER2 aptamer (HB5), while in the inner core, an ATP aptamer containing the anticancer medicine (-)-epigallocatechin gallate (EGCG) and protamine sulphate acts as a drug release switch in response to abundant intracellular ATP. The drug release investigation revealed that in the presence of 4 mM ATP, NLCs released 90% of the drug after 10 h, 50% in the presence of 0.4 mM ATP, and 40% in the absence of ATP. In the presence of 0.2% w/w sodium dodecyl sulfate (SDS), drug release from NLCS was 60% in 24 h, but it was only 15% in 24 h without SDS. They found that cellular uptake of HB5 aptamer conjugated NLCs in HER2 receptor overexpressing SK-BR-3 cells was significantly higher. There was no noteworthy difference in uptake of non-conjugated NLCs in both the cell lines. The cytotoxicity of NLCs was assessed in both cell lines in vitro. SK-BR-3 cells were 40% viable when treated with HB5 aptamer conjugated NLCs at a 30 µg/mL concentration and 50% viable with non-conjugated NLCs at the same concentration. The findings showed that aptamer attached NLCs had higher cytotoxicity due to improved targeting via HER2 receptor-mediated endocytosis. In vivo tumor inhibition was also investigated in SK-BR-7 tumor-bearing mice. Tumor inhibition was 73.33% in the group treated with HB5 aptamer attached NLCs, 65.72% in the non-conjugated NLCs group, and 54.21% in the pure EGCG group. The in vivo active targeting suggested stimuli responsiveness in tumor cells.

#### Lipid-polymer hybrid nanocarriers

Core–shell nanocarriers of polymer and lipid/lipid-PEG shells are very excellent nanocarriers for drug delivery. These NPs pose advantages of polymeric nanocarriers and liposomes, such as structural stability and biocompatibility, making them more effective for cancer targeting by fulfilling all of the compatible characteristics [91, 92]. This section discusses aptamer mediated targeting of LPHNPs for tumor treatment. Chen et al. used a self-assembly and nanoprecipitation technique to make aptamer functionalized LPHNPs for co-delivery of curcumin (CU) and carbazitaxel (CAB) [93]. For targeted distribution, they utilized the A10-3.2 aptamer (5′-GGG AGG ACG AUG CGG AUC AGC CAU GUU UAC GUC ACU CCU-spacer-NH_2_-3′ with 2′-fluoro pyrimidines). With a PDI of 0.19 ± 0.02 and a zeta potential of 23.5 ± 2.6 mV, the mean particle size of produced LPHNPS was determined to be 121.3 ± 4.2 nm. The EE of CU and CAB in the formulation was shown to be 83.5 ± 2.7% and 91.3 ± 2.8%, respectively. Similarly, the DL of CU and CAB in the formulation was also found to be 4.1 ± 0.5%, and 10.3 ± 0.8%, respectively. In 72 h, more than 85% of the drug was discharged, demonstrating that the formulations were long-lasting. Cellular uptake and dose-dependent cytotoxicity against LNCaP cells were investigated, and it was discovered that when aptamer-conjugated LPHNPs were compared to non-conjugated LPHNPs, the aptamer-conjugated LPHNPs showed significantly greater cytotoxicity (> 70%, P < 0.05). They investigated tissue distribution and pharmacokinetic aspects after providing aptamer-conjugated LPHNPs at 2 mg/kg CU and 5 mg/kg CAB doses into prostate cancer xenografted BALB/c mice. The C_max_, AUC, and t_1/2_ of CU were found to be 11.3 ± 0.9 g/mL, 216.3 ± 15.9 gm/mL hour, and 12.3 ± 0.8 h, respectively in the group treated with aptamer conjugated CU loaded LPHNPs, while aptamer conjugated CAB loaded LPHNPs, showed the C_max_, AUC, and t_1/2_ of 25.9 ± 1.6 gm/mL, 469.8 ± 33.9 gm/mL hour and 13.2 ± 1.1 h, respectively. In the group treated with CU and CAB loaded LPHNPs, the C_max_, AUC, and t_1/2_ of CU were determined to be 10.3 ± 0.7 gm/mL, 136.7 ± 9.7 gm/mL hour, and 8.5 ± 0.8 h, respectively, while CAB were determined to be 24.6 ± 1.7 gm/mL, 335.7 ± 19.6 gm/mL hour, and 7.9 ± 0.9 h, respectively. In comparison to traditional CU/CAB loaded LPHNPs, aptamer conjugated CU and CAB co-laden LPHNPs demonstrated much higher tumor suppression in vivo and data clearly indicated the improved pharmacokinetic activity of aptamer conjugated LPHNPs. Aravind et al. also reported taxane-loaded LPHNPs wherein magnetic fluid and PTX-loaded aptamer conjugated polymeric NPs were produced [94]. For targeted drug delivery, they utilized Aptamer AS1411 (NH_2_-5′-GGT GGT GGT GGT TGT GGT GGT GGT GG-3′). The synthesized NPs had a particle size of 218 nm, a zeta potential of − 27 ± 3 mV, and a PDI of less than 0.1. According to TEM and AFM pictures, the produced NPs were spherical with a smooth surface and particle sizes of 50–300 nm. The medication concentration inside the NPs ranged from 29.5% to 36.12 ± 3.5%, depending on the concentration of magnetic fluid. They used a vibrating sample magnetometer to validate the super paramagnetic behaviour of aptamer coupled magnetic fluid and PTX-loaded NPs. L929 and MCF-7 cells were used to assess the cellular absorption of produced NPs. MCF-7 cells absorbed significantly more aptamer coupled magnetic fluid and PTX-loaded NPs than cells treated with non-targeted magnetic fluid and PTX-loaded NPs. Lysotracker staining was used to observe the NPs' lysosomal localization. The dose-dependent cytotoxic impact of aptamer coupled magnetic fluid and PTX-loaded NPs was investigated for 5 days on MCF-7 cells at concentrations of 250 µg/mL, 500 µg/mL, and 1000 µg/mL. The viable cell count was determined to be around 15% after 5 days of incubation at a concentration of 1000 µg/mL, indicating that the aptamer coupled NPs were more efficient than conventional NPs. These NPs had no effect on L929 cells. Apart from actively carrying medications into particularly targeted tumor sites, these aptamers coupled magnetic NPs can also be employed to produce hyperthermia or facilitate magnetic guidance of particles to tumor regions. Chakraborty et al. also reported taxane (PTX)-loaded aptamer functionalized NPs for target-specific delivery to neoplastic hepatocytes [95]. They made NPs with several aptamers from the L1-L5 pool and discovered that aptamer L5 produced good results. The synthesized L5 aptamer-NPs had an average particle size of 211.9 ± 13.43 nm, a PDI of 0.285, and zeta potential of 15.6 ± 4.06 mv. The EE and DL were determined to be 70.95 ± 3.69 and 6.45 ± 0.45%, respectively. Hep-G2 and Huh-7 cells were used to test the cytotoxicity of L5 aptamer-NPs in vitro. Targeted L5-PTX-NPs had IC_50_ values of 42.87 ± 2.56 nm and 46.64 ± 6.48 nm against Hep-G2 cells and Huh-7 cells, respectively. Similarly, the percentage inhibition by L5-PTX-NPs against normal liver cells (WRL-68 and Chang liver) was determined to be 8.36 ± 2.91% and 8.04 ± 2.44%, respectively, indicating that targeted formulations had better distribution and targetability toward cancer cells. Incubation with L5-PTX-NPs resulted in 17.2% initial apoptotic cells and 63.8% late apoptotic cells in HepG2 cells. Huh-7 cells treated with L5-PTX-NPs showed 20.5% early apoptotic and 58.9% late apoptotic. The results of molecular docking revealed that heat shock protein 70 (HSP-70) and tumor-associated glycoprotein 72 were the most likely biomarker proteins involved for L5 Apt binding (TAG-72). Hydrogen bonding, salt bridges, attractive charges, and other interactions were all possibilities for the binding efficacy. Overall results indicated that the superior efficacy of PTX-NPL5 may be owing to L5's high affinity for neoplastic hepatocytes, resulting in maximal drug-nanocarrier penetration in them.

Powell et al. developed aptamer-functionalized LPHNPs to deliver siRNA for breast cancer treatment [96]. They used thin high-pressure homogenization and vortexing of protamine sulfate into the siRNA solution, followed by aptamer conjugation, to make LPHNPs in different batches. In the first batch (F21), aptamer and siRNA conjugated LPHNPs had an average particle size of 270 ± 10 nm and zeta potential of 32 ± 2 mV. Similarly, the second optimized batch (F31) showed mean particle size of 237 ± 12 nm and zeta potential of 31 ± 2 mV. A ribogreen test was used to measure the siRNA EE in the hybrid NPs (without aptamer tagging). The F21 and F31 both the selected batches, showed 55.25% and 64.45% siRNA encapsulation, respectively. In vitro tests were performed to access the cytotoxicity of the LPHNPs against 4T1-R mouse breast cancer cells. The F21 and F31 batches of LPHNPs containing 150 pmol siRNA exhibited 32.3% and 20% cytotoxicity, respectively, against 4T1-R mouse breast cancer cells. The in vitro cellular uptake of produced LPHNPs was investigated using a fluorescence-activated cell sorting method to determine transfection effectiveness. Aptamer attachment on LPHNPs increases 2.5 × siRNA delivery in Her-2 positive 4T1-R cells and 1.7 × in SKBR-3 cells compared to non-conjugated LPHNPs. They also discovered that siRNA-LPHNPs can knock down P-gp. In 4T1-R cells treated with aptamer conjugated LPHNPs, P-gp knockdown was obtained to be 65%, compared to 29% in cells treated with un-conjugated LPHNPs. Likewise, P-gp knockdown was shown 82% in SKBR-3 cells cultured with aptamer-attached LPHNPs, compared to 40% without aptamer conjugation. This exploratory study found that aptamer-functionalized hybrid NPs could be used to deliver P-gp specific siRNA into breast cancer cells to combat chemoresistance. Apart from breast cancer aptamer-conjugated LPHNPs have been used for the treatment of lung cancer. Wu et al. used a thin film hydration approach followed by ultrasonic dispersion to make docetaxel prodrug (DTXp) and cisplatin (DDP) co-loaded LPHNPs conjugated with A549 cell-binding aptamer for lung cancer targeting [97]. The mean particle size of prepared LPHNPs was found to be 213.5 ± 5.3 nm with zeta potential of 15.9 ± 1.9 mV. The EE and DL for DTXp were obtained at 81.2 ± 3.1% and 5.1 ± 0.9%, respectively. Similarly, the EE and DL for DDP were obtained at 82.1 ± 2.2% and 4.9 ± 0.5%, respectively. The prepared LPHNPs were stable in serum and showed sustained drug release. The DDP release was not affected in hypoxic conditions, but the DTXp release was faster in hypoxic conditions. The 50% release of both the drugs was observed initially in 8 h and more than 80% in 72 h. They used A549 and BEAS2B cells for the cellular uptake tests. In A549 cells, aptamer conjugated LPHNPs had a cellular uptake of more than 70%, while non-conjugated LPHNPs had a cellular uptake of less than 50%. There was no significant effect of aptamer conjugation on cellular uptake by BEAS2B cells. They incubated A549 cells with DTXp and DDP-loaded aptamer conjugated LPHNPs in which DTXp and DDP ratio was varied. Formulation with a ratio of 1:1 was found to be the most effective concentration. The in vivo distribution study was carried out in BALB/c mice xenografted with A549 tumor cells. The drug concentration in tumors of mice treated with aptamer conjugated LPHNPs was significantly higher than the group treated with non-conjugated LPHNPs (P < 0.05). Thus, lipid-polymer hybrid nanoparticles conjugated with aptamers cause high tumor reduction and aid in co-delivery of one or more hydrophobic/hydrophilic drugs making them crucial for targeted cancer therapy.

### Inorganic nanoparticles

The availability of a widespread range of inorganic NPs and the diversity of fabrication procedures have facilitated the creation of novel drug delivery systems. The first and most important concern of inorganic nanosystems is biocompatibility. Owing to the absence of proof and data on bio-safety, notably bio-degradation behavior, excretion pathways, and long-term toxicity evaluations, clinical use of inorganic nanoconstructs for medication administration is a major topic compared to well-developed organic nanomaterials. Extensively constructed bio-compatible inorganic materials-based nanoconstructs provide a once-in-a-lifetime chance and demonstrate the promising future of tailored therapy for a variety of ailments. Combining components of inorganic NPs with components of organic NPs has the potential to open up a new path to therapies. Researchers have created a variety of carrier systems, including metallic, nonmetallic, and metalloid nanocarriers. Gene delivery, high cellular absorption capacity, nonimmunogenic reactivity, low cytotoxicity, imaging, diagnostics, and effective targeting capability are all expected to benefit from inorganic NPs [98, 99].

#### Metallic NPs

**Fig. 7:**
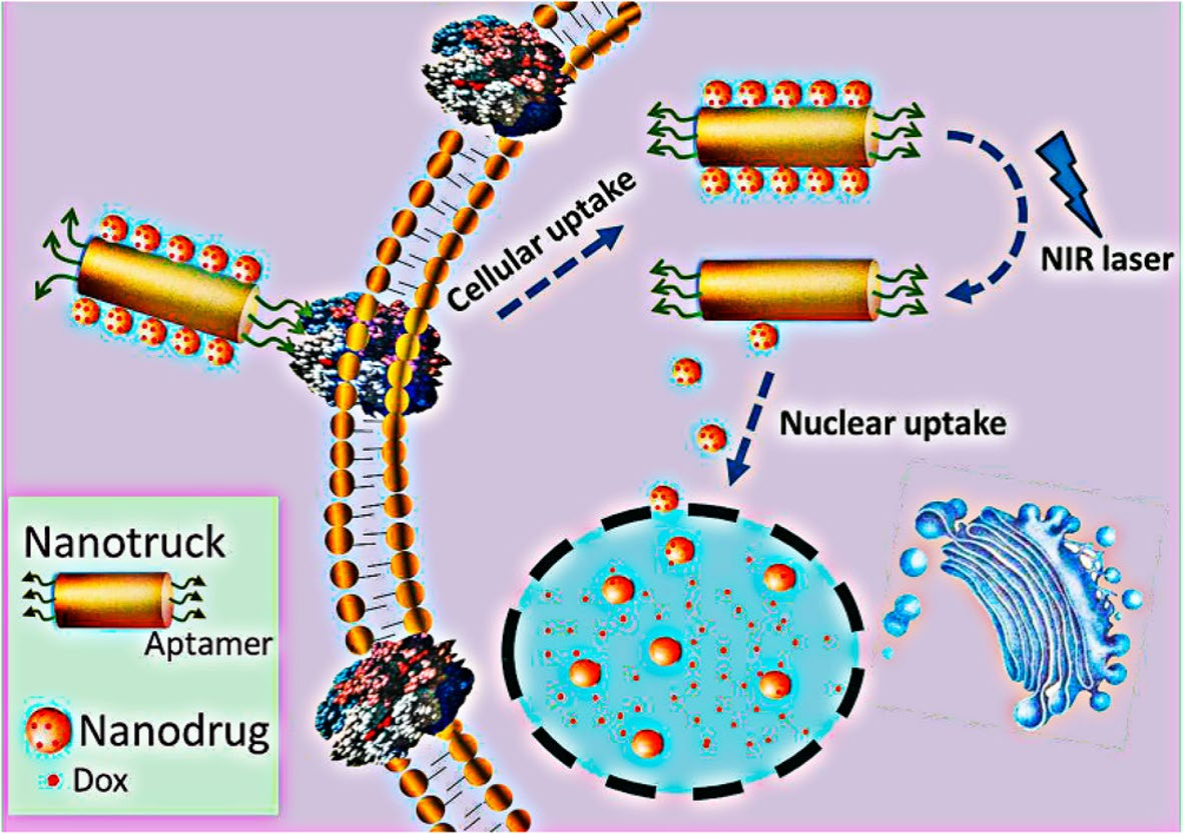
Schematic Illustration of the Cell-Targeted Photocontrolled Nuclear-Uptake Nanodrug Delivery System for Cancer Therapy. Reproduced with permission from Qiu et al. [100]. The figure is reproduced under Creative Commons Attribution License

Metallic (gold, silver, and iron-based) drug delivery systems are a major resource in nanomedicine for regulating and targeting the distribution of potential drugs and genes for greater therapeutic efficacy. This section discusses utilization of aptamer conjugated metallic nanocarriers for the treatment of cancer. Qiu et. al. synthesized NP-DOX/NR-aptamer nanocomplexes, a photocontrollable nanodrug delivery system (Fig. 7) that allows anticancer medications to accumulate in the nucleus and kill cancer cells with the help of a cell type-specific aptamer [100]. They used Sgc8 aptamer as a targeting moiety to achieve protein tyrosine kinase (PTK) mediated drug delivery. Firstly, they prepared drug-loaded gold NPs, laden on nanorods and then conjugated with aptamer to form nanoassemblies. The drug release was obtained to be 30% in 60 h. They used human acute lymphoblastic leukemia (CEM) and human Burkitt’s lymphoma (Ramos) cell lines. They also tested the aptamer's targeting capabilities against PTK positive CEM cells and PTK negative Ramos cells. In comparison to Ramos cells, CEM cells showed much increased cellular absorption of aptamer attached NPs. The IC_50_ value of CEM cells incubated with NP-Dox/NR-aptamer nanocomplexes was found 0.36 µM and that without NIR was found to be 1.22 µM. They also used the KK1B10 aptamer to target MDR K562/D cells and found that aptamer attached nanoassemblies outperformed non-conjugated nanoassemblies in terms of targeting. Overall, NIR irradiation causes nanodrugs to be released from NP-Dox/NR-aptamer nanocomplexes due to the photothermal action of the NR, which causes the DNA-duplex link between NP and NR to be broken, allowing nanodrugs to flow into cancer cell nuclei and induce death. Baneshi et al. designed AS1411 aptamer-functionalized albumin NPs laden on iron oxide (IONPs) and gold NPs (GNPs) [101]. Firstly, they prepared iron oxide and gold NPs by ultrasound and controlled the seeded growth synthesis method. The aptamer conjugated NPs of iron oxide and aptamer conjugated gold NPs of DOX were prepared by the desolvation crosslinking method. The mean particle size of prepared NPs was obtained to be 125.3 ± 20.4 nm with a zeta charge of − 50.3 mV. The TEM images showed that the prepared NPs were uniform circular shape with an average diameter of 120 nm. The loading of iron oxide and gold on albumin NPs was confirmed via TGA method by weight changes measurement. The EE and DL of DOX on NPs were found to be 63.0% and 36.5%, respectively. The MTT test was used to assess the in vitro anti-tumor activity of the proposed delivery vehicle on MCF7 and SKBR3 human cancer cells with nucleolin receptors. The formulation showed only ~ 55% in vitro drug released in 10 days in PBS at pH 7.4. The degree of early and late apoptosis produced by aptamer attached NPs against MCF-7 cells was obtained at 6.7% and 16%, respectively, compared to 5.4% and 9.3% with non-conjugated NPs. Furthermore, GNPs and IONPs coated with albumin showed no toxicity. The results showed that AS1411 aptamer-functionalized NPs increased absorption and effectiveness in MCF7 breast cancer cells compared to non-targeting NPs due to the significant aptamer affinity for the overexpressed nucleolin on the cell surface.

Alijani et al. prepared iron oxide metal–organic framework (MOFs) of DOX for target-specific treatment of triple-negative MDA-MB-231 breast cancer cells [102]. Initially, they prepared iron oxide MOF by ultrasonication. Then they conjugated DOX with MOF and after that carbon dots were conjugated with the same MOF followed by AS1411 Apt with MOF of DOX and carbon dots. The prepared NPs were around 16 nm in size, monodispersed, and smooth surfaced. The DOX loading was found at 87.77%. The in vitro drug release was obtained higher in acidic pH as compared to in basic pH. The drug release after 4 days in pH 5.5 was 47.3% and that in pH 7.4 was 29.7%. The in vitro cytotoxicity of prepared NPs was evaluated against MDA-MB-231 cells. The cells were treated with aptamer conjugated MOFs loaded with DOX and carbon dots, iron oxide MOFs loaded with DOX and carbon dots, and lastly, iron oxide MOFs with DOX were found to have 23%, 32%, and 39% viability, respectively. The aptamer conjugated formulation showed more significant cytotoxicity and target-specific delivery through nucleolin receptors over expressed on MDA-MB-231 cells. The aptamer AS1411 showed good attachment and targeting ability to nucleolin receptors of MDA-MB-231 cells. Shiao et al. used photosensitizer, 5, 10, 15, 20-tetrakis (1-methylpyridinium-4-yl) porphyrin (TMPyP4), which were employed to create aptamer (AS1411) linked photoresponsive gold NPs for targeted administration of DOX [103]. The mean particle size of prepared NPs was obtained at 40.1 ± 1.4 nm with a zeta potential of -39.4 ± 1.6 mV. The DOX and TMPyP_4_ loading was obtained to be 81 ± 7% and 87 ± 4%, respectively. The in vitro cellular uptake of prepared NPs was studied in HeLa cells using atomic absorption spectrometry (AAS). The cellular uptake of aptamer conjugated NPs was found at 1.5 ± 0.3 × 10^5^ NPs per cell. In contrast, the non-conjugated NPs showed less than 6.1 ± 0.9 × 10^4^ NPs per cell. The in vitro cytotoxicity study of prepared NPs showed that the aptamer conjugated DOX and TMPyP_4_ loaded NPs were 2.5-fold more effective than photodynamic treatment alone and 4.6-fold more effective than pure DOX against HeLa cells. The photodynamic and drug treatment showed a synergistic increase in the anticancer effect. The cytotoxic effect of aptamer conjugated NPs was also studied by determining ROS production after treatment. The HeLa cells incubated with aptamer conjugated DOX and TMPyP_4_ loaded gold NPs showed 1.6-fold higher effectiveness than cells incubated with non-conjugated DOX and TMPyP_4_ loaded gold NPs. For the comparative study, they treated MDR MCF-7R cells, and MCF-7 cells with the same concentration of aptamer conjugated DOX and TMPyP_4_ loaded gold NPs and then determined the up-regulation of P-gp. The P-gp up-regulation in MCF-7R cells was found to be 12-fold higher than in MCF-7 cells after the same treatment. This high increase in P-gp clearly indicated that the prepared NPs were more effective against MDR MCF-7 cell line. The results indicated that the aptamer conjugation enhanced cellular uptake by nucleolin mediated endocytosis.

Cai et al. (2020) prepared AS1411 Apt functionalized molybdenum disulfide nanosheets of DOX with polydopamine (PDA) by ultrasonication method, which showed lysosomal microenvironment/NIR responsive synergistic chemo-photothermal therapeutic effect [104]. The particle size of prepared pegylated Molybdenum disulfide of DOX with PDA layer nanosheets (Apt-PEG-PDA-MoS_2_-DOX) was around 150 nm diameter and 8 nm thickness with flaky shaped morphology. The zeta potential of nanosheets was found -45 mV. The EE and DL of DOX were found 73.40% and 3.85%, respectively. The drug release without laser irradiation after 72 h was found less than 20% at pH 7.4 and ~ 50% at pH 5, which indicated faster release in an acidic medium. The percent hemolysis caused by Apt-PEG-PDA-MoS_2_-DOX at a concentration of 200 µg/mL after 24 h of incubation was found to be less than 5% which indicated the biocompatibility of nanosheets. They also looked at the cytotoxicity of 808 nm NIR on MCF-7 cells and found that it did not cause any major harm. The capability of Apt-PEG-PDA-MoS2-DOX to target MCF-7 and MCF-10A cell lines was tested in vitro. Due to overexpressed nucleolin receptors, the cellular absorption of Apt-PEG-PDA-MoS2-DOX was substantially higher in MCF-7 cells than in MCF-10A cells. The MCF-7 cells treated with Apt-PEG-PDA-MoS2-DOX in the presence of NIR 808 nm light displayed DOX fluorescence inside the nucleus, but following the same treatment without NIR irradiation, fluorescence was only found in the cytoplasm. Likewise, MCF-7 cells were killed by Apt-PEG-PDA-MoS2-DOX without NIR 808 nm at 32.1% and with Apt-PEG-PDA-MoS2-DOX NIR 808 irradiation at 91.8%, indicating a synergistic impact of DOX and NIR irradiation. These results clearly indicated that the aptamer AS1411 targeting of drug to MCF-7 cells through nucleolin receptors.

**Fig. 8:**
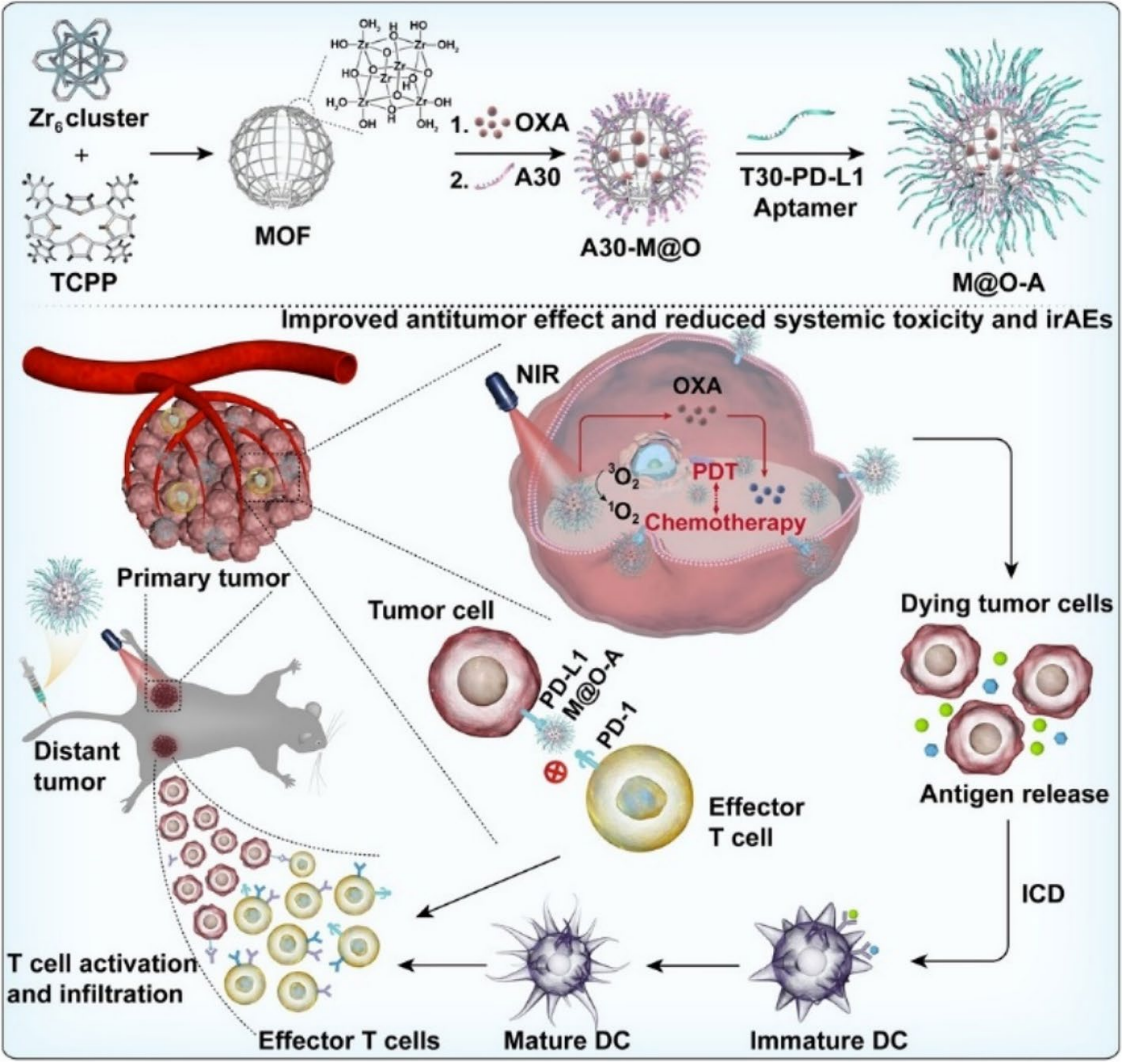
Schematic illustration of the design and synthesis of M@O-A NPs to enable concurrent photodynamic therapy, controlled released chemotherapy, and primed cancer immunotherapy with enhanced safety. Reproduced with permission from Zhang et al. [105]. Copyright John Wiley and Sons (2022)

Zhang et al. mention immune checkpoint blockade as a paradigm-shifting treatment modality. The nanosystem is responsive towards photodynamic therapy, chemotherapy, and enhanced immunotherapy [105]. As shown in Fig. 8, zirconium based PCN-224 commenced with a metal–organic framework core loaded with oxaliplatin and having a shell of aptPD-L1. Using TEM and energy-dispersive X-ray elemental mapping it was observed that Zr (MOF), Pt (Oxaliplatin), and P (DNA) was overlapping showing successful loading of OXA in the MOF and conjugation of Apt on the loaded nanocarrier. Furthermore, a change in zeta potential was observed from positive to negative on conjugating the aptamer, along with an increase in hydrodynamic radius. The production of singlet oxygen species by MOFs was evaluated by the singlet oxygen sensor green (SOSG) assay. The fluorescence increased in the MOF NPs suspension when irradiated with 640 nm laser, while fluorescence remained unchanged without irradiation. For evaluating the targeting ability of the nanosystem, PD-L1 overexpressing cell line MC38 were treated with the aptamer-linked nanoparticles, alongside mutated aptamer and preincubated PD-L1 antibody. The fluorescent signal was much higher from the aptamer-linked nanoparticle in comparison with the other two groups. When the effect of M@O-A NPs was evaluated on cells, almost all the cells underwent apoptosis upon 640 nm irradiation. The group further investigated the possibility of cell undergoing immunogenic cell death (ICD) by checking for the specific cell surface marker calreticulin (CRT). The cell treated with M@O-A and radiation showed a strong red fluorescence in comparison with the non-irradiated group. The in vivo biodistribution assessment showed that on intravenous injection the nanoparticles accumulated in the tumor and then decreased over time. The in vivo anti-tumor activity was assessed in a total of 12 groups including PBS, free drug, free aptamer, and nanoparticles with and without subjecting the animals to irradiation. Among all the groups the M@O-A NPs subjected to 640 nm irradiation showed the best therapeutic efficacy and longest survival. Since anti-PD-L1 antibody shows immune-related adverse effects (irAEs) in cancer models, the researchers wanted to evaluate irAEs related to the aptamer-linked nanoparticles. A special clinical irAEs transgenic mouse model was established and treated with PD-L1 antibody, aptamer NP-conjugate, and diphtheria toxoid. The mice were sacrificed on day 18 and histological screening of the organs showed major pathology changes in colon, liver, kidney, and spleen with significant damage to other tissues. on the other hand, MOF@O-A(+ R) did not show any histological toxicity and the scores were much less in comparison with PD-L1 antibody and DT. The study provides a photodynamic nanosystem capable of immune suppression for cancer eradication and eliminating immune-related adverse effects (irAEs) associated with use of antibodies.

Similarly, for nucleolin receptor-mediated DOX targeting MCF-7 cells, Su et al. created aptamer-templated silver nanoclusters embedded in a zirconium metal–organic framework (Zr-MOF) [106]. The group synthesized UIO-66 (Universitetet i Oslo), a metal–organic framework by hydrothermal method. After that, the aptamer conjugated silver nanoclusters (Zr-MOF) loaded with DOX (Apt-DOX UiO-66-AgNCs-NCs) were prepared by one-pot encapsulation method. The SEM images of prepared NCs showed a spherical shape with a size of 4–8 nm, and with a DOX loading of 46.7%. The drug release was 30.3% in pH 5 and 14.4% in pH 7.4, and after 96 h, the drug release was 44.3% in pH 7.4 and 57.2% in pH 5, indicating that the overall release was faster in acidic pH. The in vitro targeting ability and cytotoxicity of NCs were evaluated against L929 and MCF-7 cells. The AS1411 Apt attached NCs were quickly taken up by both cells via nucleolin receptor mediated endocytosis, and cytotoxicity studies revealed that the AS1411 Apt was more selective for MCF-7 cells than L929 cells. In the case of MCF-7 cells and L929 cells, the percent of cells killed by Apt- DOX UiO-66-AgNCs-NCs at a concentration of 10 µg/mL was found to be 80.3% and 54.9%, respectively. Chen et al. also reported ATP-responsive, AS1411 Apt and Rhodamine 6G coupled metal organic framework (MOF) based NPs for targeted drug delivery [107]. The NPs were synthesized by the click chemistry technique. The BET measurement showed that the NPs have 1100 m^2^.g^−1^ surface area with a pore size of 1.54 nm. The in vitro release of Rhodamine 6G in the presence of 25 × 10^−3^ m ATP (biomarker, overexpressed in cancer cell) reached saturation in 45 min. The DOX-loaded NPs also showed the complete release of DOX in 1 h in the presence of 25 × 10^−3^ m ATP. The cellular uptake of AS1411 aptamer conjugated NPs was much higher in MDA-MB-231 cells in comparison to the uptake in MCF-10A cells. Similarly, the cytotoxicity of the aptamer conjugated NPs against MDA-MB-231 cells was much greater than that of non-conjugated NPs in the same cell lines. According to these findings, the aptamer AS1411 was shown to be unique to MDA-MB-231 cancer cells and did not cause toxicity in normal MCF-10A breast epithelial cells.

**Fig. 9:**
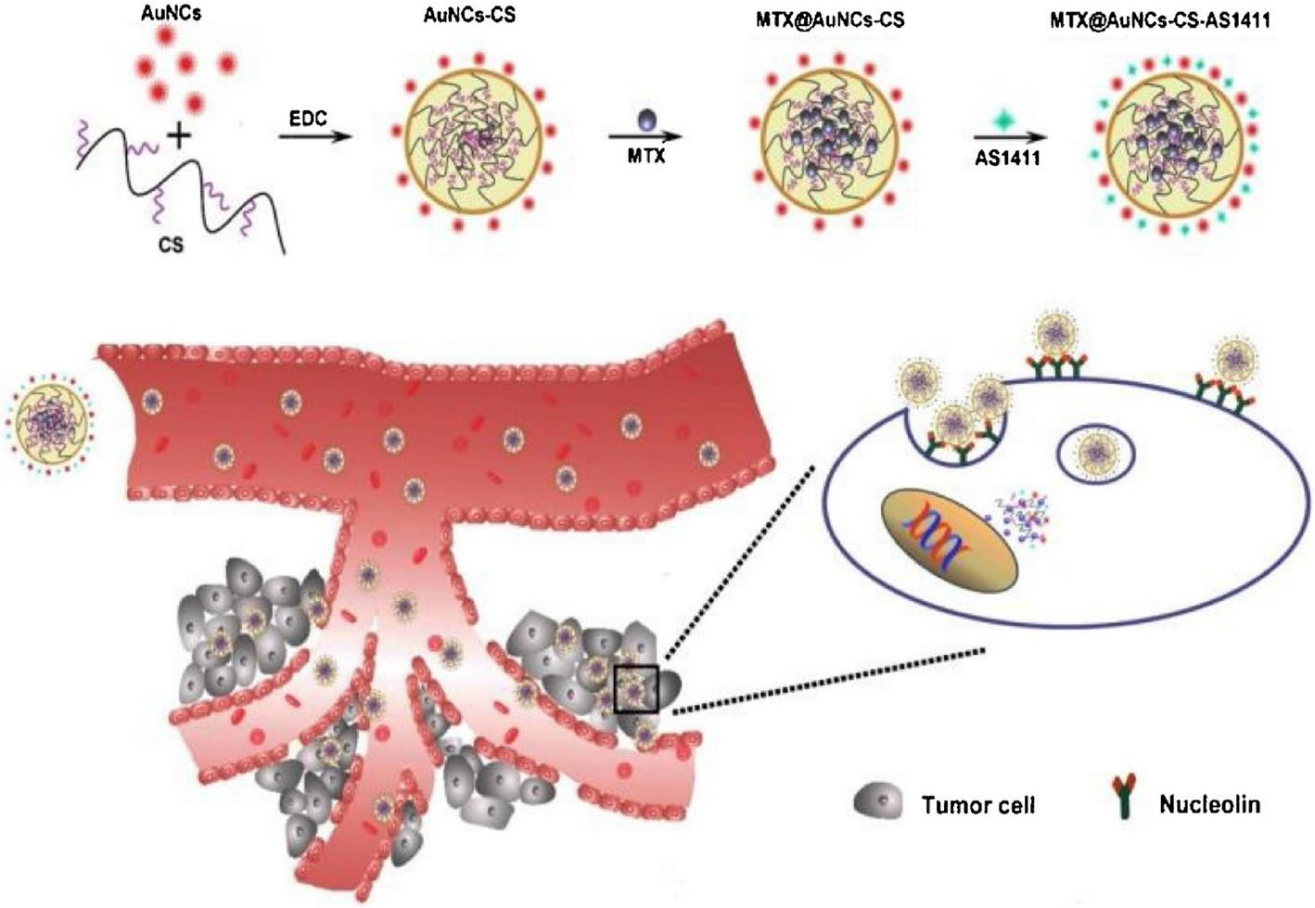
A schematic illustration of the synthetical route of MTX@AuNCs-CS-AS1411, and the proposed blood circulation and intracellular uptake and drug release. The actively targeted nanoparticles can be released in a controlled manner, achieving enhanced targeting efficacy by active targeting to individual tumor cell and deep penetration within the tumor tissue. Reproduced with permission from Guo et al. [109]. Copyright Elsevier (2018)

Roy et. al. prepared locked nucleic acid (LNA) aptamer based multi-modal iron oxide saturated lactoferrin (LF) nanocarriers (NCs) [108]. The particle size of prepared nanocarriers was found to be 250–300 nm. The anti-cancer activity of prepared nanocarriers was evaluated on CD133, EpCAM and CD44 positive Caco-2 cells xenografted mice. The mice were fed with an AIN93G diet containing nanocarriers. They observed 6.8-fold and 2.91-fold reduction in tumor volume in the group treated with non-conjugated nanocarriers and aptamer conjugated nanocarriers, respectively. Tumor regression was found in 70% of mice fed with non-targeted NCs, with 30% of mice displaying tumor recurrence after 30 days, whereas tumor recurrence was observed in only 10% of mice fed with targeted NCs, indicating a considerably greater survival rate. The anti-angiogenic effect of prepared NCs was evaluated by measuring the expression of the apoptotic protein in mice. Mice treated with aptamer conjugated NCs showed increased protein expression of Bad, Bcl-2, catalase, clusterin, survivin, and XIAP by 1.6, 2, 2.5, 1.5, and 2.6 times, respectively, as compared to untreated mice. Pre-apoptotic markers including Bax, pro-caspase-3, cytochrome C TRAIL, FADD, HSP-27, HSP-60, HSP-70, HTRA2 phospho-p53 (S15), phospho-p53(S392), and SMAC/Diablo were also raised by 2, 4.6, 4, 2, 1.5, 6.6, 2.2, 4, 6, 2, 17 and sixfold, respectively, in mice treated with aptamer coupled NCs. After treatment with various nanocarriers, they also looked at CD133, EpCAM, and CD44 cell expression in mouse tumors. There was no expression of CD133, EpCAM and CD44 in tumors of mice treated with Apt conjugated nanocarriers were high as expression of these 3 cells was observed in untreated group. The group treated with non-conjugated nanocarriers showed lower expression of CD133 and EpCAM and no expression of CD44 cells in tumor. Apart from the promising anti-cancer efficacy and exceptional tumor-targeting ability, these NCs also maintained immunomodulatory benefits in mice by increasing RBC, hemoglobin, iron, calcium, and zinc levels. Guo et al. prepared AS1411 aptamer conjugated chitosan-gold hybrid NPs of MTX for the treatment of lung cancer (Fig. 9) [109]. The mean particle size of prepared NPs was obtained at 200 ± nm with a zeta potential of -0.42 mV. At pH 5.5, drug release was 70% in 2 h and 93% in 24 h; at pH 7.4, drug release was 40% in 2 h and 76% in 24 h. The in vitro cellular uptake of prepared NPs was evaluated against A549 and LO2 cell lines. The Apt conjugated NPs showed much higher cellular uptake in A549 cells, whereas only small quantity of NPs were taken up by LO2 cells, which showed aptamer specificity towards A549 cells. The in vitro anticancer effect of NPs was evaluated against A549 cells. The percentage apoptosis after treatment with free MTX, MTX loaded gold-chitosan hybrid NPs and AS1411 aptamer conjugated MTX loaded gold-chitosan hybrid NPs was found 31%, 36% and 42%, respectively. The tumor weight in the group treated with Apt conjugated MTX NPs, MTX NPs, Free MTX, placebo gold-chitosan NPs and normal saline were obtained at approximately 60, 85, 160, 290 and 310 mg, respectively. These results indicated the capacity of produced NPs to target tumors in vivo and their higher anti-tumor impact in vivo than non-conjugated NPs. There was no substantial weight loss in the group treated with blank NPs at a concentration of 600 µg/mL, indicating the in vivo safety of NPs.

**Fig. 10:**
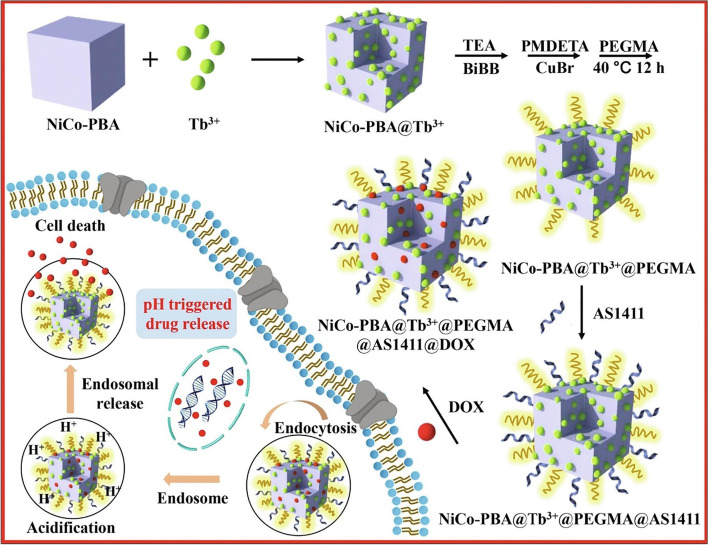
Schematic illustration of the synthetic procedure for core–shell-structured NiCo-PBA@Tb^3+^@PEGMA@AS1411 composite and NiCo-PBA@Tb.^3+^@PEGMA-@AS1411- based DOX loading and pH-responsive controlled release system. Reproduced with permission from Jia et al. [111]. Copyright Elsevier (2020)

Chen et al. developed two species of prussian blue based-nanoparticles (PBNPs) having a metal–organic framework using cobalt (cobalt-iron) and copper (copper-iron) for the delivery of doxorubicin [110]. The cuboid NPs were then modified with polyethylene glycol methacylate (PEGMA) followed by attachment of the aptamer AS1411. Both species of PBNPs showed more than 80% dox loading efficiency with higher release of 75.9% observed in copper-iron based PBNP. These were evaluated for their cellular internalization, in vitro toxicity and in vivo localization. Copper iron PBNPs showed higher in vivo targeting and tumor size reduction than cobalt-iron PBNPs. Thus, the developed nano-drug delivery system can be an effective for breast cancer chemotherapy. Similarly, Jia et. al. synthesized PEGMA-modified bimetallic NiCo Prussian blue equivalent doped with terbium (Tb) (III) ions and conjugated them with AS1411 Apt (Fig. 10) to achieve target-specific delivery and pH-responsive release of anticancer drug [111]. The DLS results showed that the mean particle size of NiCo-PBA-Tb^3+^ was 172 nm, and DOX loading efficiency was obtained to be 60.3%. The in vitro drug release from the aptamer-PEGMA modified nanoparticle was obtained to be 18.2%, 30.7%, and 40.5% at different pH (5, 6.8, 7.4 respectively) condition within 8 h, it showed the faster drug release in acidic pH as compared to neutral. The in vitro cellular uptake and cytotoxicity study was determined against L929 and 4T1 cells. DOX-loaded NiCo-PBA-Tb^3+^-PEGMA-AS1411 had a substantially greater cellular absorption in 4T1 cells than DOX-NiCo-PBA-Tb^3+^-PEGMA formulation in L929 cells. The placebo effect of aptamer conjugated NPs at 200 µg/mL concentration showed slight cytotoxicity towards L929 and 4T1 cells. The DOX loaded NiCo-PBA- Tb^3+^-PEGMA-AS1411 was more cytotoxic as compared DOX loaded NiCo-PBA- Tb^3+^-PEGMA as evaluated against 4T1 cells. These results revealed that higher anticancer efficacy of the drug could be achieved by preparing target-specific drug delivery system using aptamers. The in vivo antitumor effect of nanocomposite was studied in 4T1 cells induced tumor in BALB/C mice. The tumor volume of the group treated with normal saline was increased by 2.5-fold after 14 days. The group treated with DOX-loaded NiCo-PBA-Tb^3+^-PEGMA-AS1411 showed a slow increase in tumor volume in the initial 4 days, rapid increase in 6 days and steady decrease from 8^th^ day of treatment. The in vivo antitumor effect indicated that the aptamer conjugated DOX-loaded NiCo-PBA- Tb^3+^-PEGMA showed enhanced anticancer activity.

Zhang et al. also synthesized smart Cu (II)- Chlorin e6 (Ce6) labeled TLS11a aptamer complexes and prodrug Banoxantrone (AQ4N) conjugated based gold nanoplatform for treatment of liver cancer [112]. The mean particle size of prepared NPs was obtained at 137.07 ± 4.26 nm with a PDI of 0.142 and zeta potential of -32.6 ± 1.1 mV. The drug release at pH 4.5 was higher (65.17%) in 1 h and reached 74.17% in 12 h. The released Cu (II) concentration was found to be 70.11 M, or 75.19% of total added Cu (II), indicating that NPs released more drug in the acidic pH of the tumor microenvironment, as demonstrated by the color change from red to blue. The in vitro cellular uptake study revealed that the aptamer conjugated NPs showed much higher cellular uptake in HepG2 cells compared to HeLa cells which indicated the specificity of TLS11a aptamer towards HepG2 cells. The in vitro synergistic effect of AQ4N-CU (II)-APT-GNPs and 670 nm laser ON/OFF cycling in 700 s intervals were evaluated by CLSM study. They discovered that combining AQ4N-CU (II)-APT-GNPs with a 670 nm laser for 5 min produced more ROS than AQ4N-CU (II)-APT-GNPs alone, demonstrating the combined NPs and laser synergistic anticancer impact. The biocompatibility of prepared NPs was evaluated against normal LO2 cells. They found that the viable cell count was more than 85%, showing the prepared NPs were safe for biological use. The capacity of aptamer attached NPs to kill HepG2 and HeLa cells in hypoxic conditions was substantially higher than in the presence of oxygen, indicating a greater tumor inhibitory impact.

#### Non-metallic-nanostructures and metalloids

Carbon, graphene, phosphorus, silicon, selenium, germanium, and arsenic are among other non-metallic/metalloid chemicals that have emerged as a growing sector, primarily for the development of diagnostic agents and drug delivery vehicles for cancer therapy. In cancer-specific theranostics, a precise chemical functionalization is combined with stimulus qualities and targeted moiety to create unique responsive nanocarriers with distinct functionality and additional advancement. Few examples of such targeted non-metallic and metalloid nanocarriers are discussed in this section.

MUC-1 aptamer functionalized magnetic graphene oxide nanocarriers for MCF-7 cell targeted delivery of PTX were produced by Hussein et al. [113]. The nanoparticles were synthesized using four different concentrations of Fe(acac)_3_ mixed with graphene oxide. The nanoparticles were then conjugated with the aptamers and characterized using UV–Visible spectrometry, FTIR, Raman, XRD, and TEM. The prepared MGO4 NPs had a maximum of 95.75% drug EE and a 19.38% DL, with a 32.50 emu/gm magnetic saturation. The cumulative medication release from MGO1 was 67.59%, 38.38%, and 17.89% at pH 2.0, 5.5, and 7.4 showing a pH responsive behavior in drug release. The biocompatibility of the generated MGO4 NPs was tested using L-929 cells. After being cultivated with 100 µg/mL, the L-929 cells were 95.82% viable, demonstrating that the NPs were biocompatible. The ability of these NPs to target MCF-7 and L-929 cells was determined using flow cytometry. When treated with conventional NPs, both cells produced the same fluorescence signal, however MCF-7 cells treated with aptamer linked NPs produced more fluorescence than L-929 cells. The cytotoxicity of generated NPs against the MCF-7 cell line was dose-dependent. According to the cytotoxicity data, MCF-7 treated cells with free PTX at a dose of 3.125 ng/mL were 17.37% viable, but MCF-7 treated cells with PTX NPs at the same dosage were 48.47% viable. They concluded that the AS1411 aptamer might be employed to transport nanocarriers to MCF-7 cells for a stronger anticancer effect. Zavareh et al. prepared a combination delivery system of chitosan/carbon quantum dots (QDs)/aptamer by emulsification method for 5FU delivery [114]. They used a 25-mer aptamer named 5TR1 to deliver 5FU to the MCF-7 cell line via MUC-1. The nanocomplex has an average diameter of 122.7 nm and zeta potential of 31.2 mV. The EE and DL were obtained to be 84.7 ± 5.5% and 37.2 ± 3.8%, respectively. In an acidic environment, the medicine was released at a faster rate. At pH 5.4, drug release was 71%, but at pH 7.4, drug release was only 23% after 24 h. MCF-7 cells had a 47% cell survival after 48 h of treatment with an aptamer conjugated nanocomplex at a 5FU concentration of 4.8 µg/mL, compared to 67% with a non-conjugated nanocomplex at the same concentration and treatment duration. According to these findings, the increased cytotoxicity of the aptamer coupled nanocomplex was owing to the high affinity and site-specific delivery. They also assessed the Bcl-2 and Bax gene expression after treating MCF-7 cells with an aptamer attached nanocomplex and discovered that the aptamer attached nanocomplex group had a lower Bcl-2/Bax ratio than the traditional nanocomplex. For target specific delivery, Savla et al. developed DOX-loaded and MUC-1 DNA aptamer conjugated QDs [115]. A hydrazine bond was used to bind DOX to QDs. The absorbance was used to determine the drug loading on QDs. Each QD had about 46 DOX molecules attached to it. The drug release was determined using the Forster resonant energy transfer (FRET) method. The drug release from the conjugate was obtained to be 35% in 5 h in an acidic environment. In vitro cellular absorption in A2780/AD cells was investigated using CLSM. MUC1 aptamer coupled DOX-QDs had a significantly greater cellular absorption than non-conjugated DOX-QDs. The FRET results demonstrated that the aptamer MUC1-QDS-DOX combination could increase cellular uptake and DOX release from QDs inside the cell, resulting in higher anticancer activity. This conjugate's cytotoxicity was tested on the DOX-resistant A2780/AD cell line. After treatment with MUC1 aptamer attached DOX QDs, the IC_50_ value was considerably reduced. This study found that drug-resistant cancer may be treated by constructing a complex and delivering it to a specific place, using an aptamer as a targeting moiety.

Jiang et al. used a solvent exchange technique to create a carrier-free nano delivery system for aptamer-specific targeting of ursolic acid (UA) and DOX to HER2 receptors [116]. NPs had a particle size of 108.9 nm and a PDI of 0.186 with negative zeta potential. In NPs, the DL of UA and DOX was found to be 80.98% and 18.56%, respectively. The produced NPs were stable at pH 7.4, but unstable at pH 6.5 and 5, and displayed rapid release. At pH 7.4, the initial burst release of both medicines was observed to be about 20%. After that, there was a sluggish release of just 25% in 12 h. Cellular uptake data showed that BT474 took up aptamer conjugated NP significantly higher than non-conjugated NPs. The results demonstrated that non-conjugated NPs induced 70.2% apoptosis, but aptamer conjugated NPs caused 81.7% apoptosis. Aptamer-conjugated NPs demonstrated a much better anticancer effect than non-conjugated NPs against the HER2 receptor overexpressing BT474 tumor. Zong et al. created a PEGylated nanosheet based on black phosphorus for chemothermal treatment of acute lymphoblastic leukemia [117]. The produced nanocarriers had a particle size of 190 nm and zeta potential of -29.9 mV with a 25% DL. Sgc8 aptamer was used to target CCRF-CEM cells through the tyrosine kinase 7 receptor. The generated PEGylated BP NPs displayed good pH and photothermal dual-responsive drug release capabilities and remarkable photothermal conversion efficiency and stability. The pH-dependent drug release of nanocarrier after 90 min at pH 7.4 and at pH 5.0 was found 9.86% and 22.34%, respectively. After 90 min of NIR illumination, drug release was determined to be 36.17% at pH 7.4 and 55.68% at pH 5, representing that NIR laser illumination enhanced the release of drugs. The toxicity of nanocarriers was tested in zebrafish embryos, and it was discovered that embryos survived at a rate of more than 85% following a 24-h treatment with nanocarriers, showing the drugs' benevolent nature. The cytotoxicity of nanocarriers was examined against CCRF-CEM cells. The number of viable cells in cells treated with aptamer conjugated nanocarriers under NIR light was reduced by 20%. As a result, the suggested BP NS-based multifunctional nanomaterial has the potential to boost curative efficiency by allowing for selective and synergetic chemophotothermal therapy of acute lymphoblastic leukemia. Zhang et al. prepared MUC-1 aptamer anchored mesoporous carbon NPs (MCN) as a bio-triggered targeted drug delivery by hydrothermal method [118]. These carbon NPs was then crosslinked with disulfide bond and then capped with polyacrylic acid to get PAA-ss-MCN-DOX. The zeta potential of prepared NPs after aptamer conjugation was obtained to be -6.6 ± 0.1 mV with 27.2% DL. The effect of GSH on drug release was studied in 20 mM GSH at pH 5.5 and obtained to be 60% in 48 h. This indicated that the prepared NPs were pH-sensitive and showed bio-triggered drug release. The DOX-loaded MCNs showed dosage-dependent cytotoxicity, which was evaluated against HeLa cells. The DOX-loaded MCNs were more cytotoxic towards GSH pre-treated HeLa cells as compared to cells without pre-treatment with GSH. The in vitro cellular uptake of prepared MCNs were evaluated against MUC1-positive A549, MCF-7 and MUC1-negative HepG2 cell lines. In the A549 and MCF-7 cells, the cellular uptake of aptamer conjugated DOX-loaded MCNs was much higher than normal DOX-loaded MCNs. In HepG2 cells, the cellular uptake of DOX-loaded MCNs was higher than aptamer conjugated DOX-loaded MCNs. The cellular uptake study clearly showed that the MUC-1 Apt was more specific towards MCF-7 and A549 cells and did not increase uptake in MUC-1 negative HepG2 cells. HepG2 cells treated with aptamer conjugated DOX loaded MCNs at a DOX dosage of 100 µg/mL had a viable cell count of 75%, whereas A549 and MCF cells had a viable cell count of 55%, respectively. The results exhibited that the MUC-1 aptamer can be used as a targeting moiety for MUC-1 positive cancer cells to achieve targeted drug delivery and better anticancer effect.

**Fig. 11:**
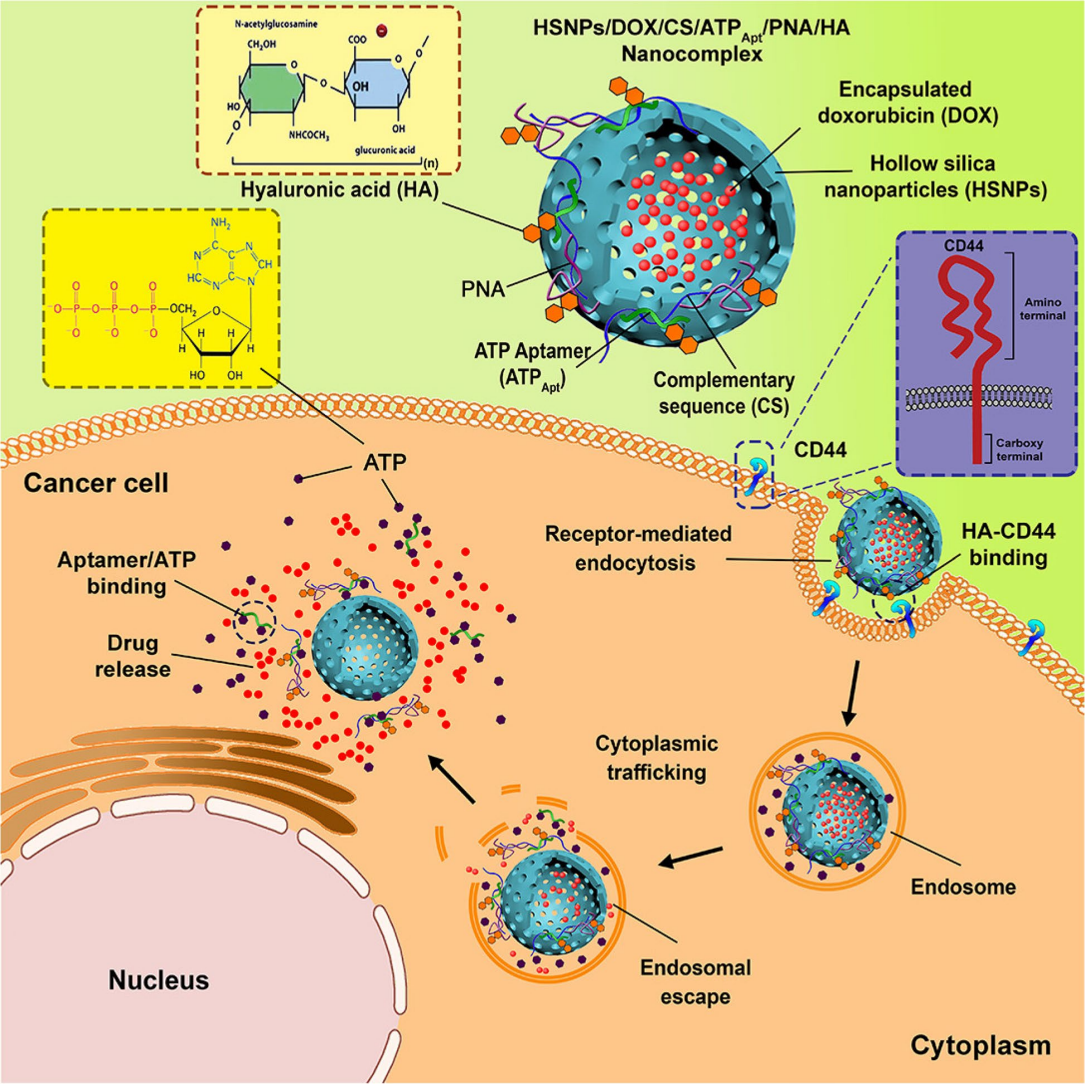
Schematic illustration of HA-targeted HSNPs modified with PNA/ATPApt as gatekeeper for the delivery of DOX. Reproduced with permission from Kazemi et al. [119]. Copyright Elsevier (2022)

Kazemi et al. developed hollow silica nanoparticles (HSNPs) for targeted doxorubicin delivery using hyaluronic acid for breast cancer therapy [119]. Within the cell, the peptide nucleic acid (PNA) and ATP aptamer were used as gatekeepers from releasing the drug. Release of drug is triggered only when the aptamer binds to ATP, opening the pore on the nanoparticle for drug to leak out. The difference between intracellular and extracellular ATP concentration is the critical point for ATP mediated cargo release. The hollow silica nanoparticles were synthesized by first making a solid core follow by a shell and then etching of the core resulting in hollow silica nanoparticles (HSNPs) (Fig. 11). The nature of HSNPs was confirmed using TEM and SEM followed by FTIR to confirm functionalization. The entrapment efficiency of DOX was approximately 33% and release at pH 7.4 was only 16% during 120 h whereas at pH 5.5, approximately 20% was released in 12 h which then shifted to stagnant release showing a total release of 38% at pH 5.5. DOX release from ATP-Aptamer gated nanocarrier was checked at 1 μM or 10 mM ATP concentration showing ~ 4% and ~ 72% release, respectively at pH 5.5 during 120 h. Similarly, at pH 7.4 the release was ~ 4% at 1 μM (ATP) as compared to 35% at 10 mM (ATP) over 120 h. Thus, showing high efficiency of the ATP-aptamer gating. Cellular uptake was checked using 4T1 and MCF-10A cells using flow cytometry which showed higher uptake of HA conjugated nanocarrier in 4T1. The viability of 4T1 was significantly decreased as compared to MCF-10A which did not show significant change. In vivo assessment showed significant decrease in tumor size. Biodistribution showed that as compared to free Dox and non-targeted nanocarriers, targeted nanocarriers had a higher accumulation of DOX in tumor. Thus, the study shows that aptamer targeting can be done to small molecules like ATP for purposes other than cell targeting.

Zheng et al. created smart nanocomposites of aptamer/DOX/graphene QDs capped with fluorescence resonance energy transfer (FRET) MSNPs for real-time monitoring of ATP-triggered drug release using a microwave-assisted irradiation technique [120]. The QDs nanocomposite capped MSNPs were then conjugated with the ATP aptamer and Aptamer AS1411. The produced QDs with aptamer conjugation had a diameter of 79 nm and were biocompatible when tested against HeLa cells. Any external or endogenous shift in ATP concentration has shown to cause a change in drug release as well as a concomitant modification in the nanocomposites signal. According to CLSM pictures and flow cytometry, the cellular absorption of aptamer attached nanocomposites was considerably greater than regular nanocomposites. NIH 3T3 and HeLa cells were used to test the cytotoxicity of produced nanocomposites. In both cell lines, the normal formulation was equally hazardous, but aptamer-linked nanocomposites were more lethal in HeLa cells than in NIH 3T3 cells because of the greater number of receptors overexpressed for AS1411 aptamers in HeLa cells. The viable cell count of HeLa cells after treatment with aptamer conjugated nanocomposites at a 20 g/mL concentration of DOX for 48 h was found to be 45%. In contrast, it was obtained to be 65% after treatment with normal nanocomposites at the same concentration of DOX and similar incubation time. As a result, the suggested multifunctional ATP triggered FRET-nanocarriers might be used for a variety of drug-release monitoring, drug transport, and cancer treatments. Shen et al. prepared pH-responsive anti-HER-2 aptamer conjugated β-cyclodextrin capped MSNPs of DOX (HAPtNPs) [121]. The mean particle size of prepared NPs was found to be 218.2 ± 6.1 nm with PDI of 0.263 ± 0.014 and zeta potential of − 31.3 ± 1.4 mV. The DL of the prepared formulation was obtained at 3.64%. The pH-dependent in vitro drug release from NPs was accessed and the results showed 19.93 ± 3%, 18.92 ± 4% and 82.3 ± 5% at different pH conditions of 7.4, 6.4 and 4.5, respectively. The in vitro cellular uptake of prepared NPs was determined against HER-2 receptor overexpressed SKBR3 cells and HER-2 receptor less expressed MCF-7 cells. The cellular absorption of HAPtNPs in SKBR3 cells was 82.7% compared to 31 percent in MCF-7 cells. SKBR3, MCF-7, and HEK-293 T cells were used to test the in vitro cytotoxicity of generated placebo NPs. In both MCF-7 and HEK-293 T cell lines, the placebo NPs exhibited no cytotoxicity. However, in SKBR3 cells, cell viability was only 55% at a 500 g/mL dose. The results clearly indicated that the placebo HAPt conjugated NPs exert potent cytotoxicity against HER-2 receptor overexpressed cells. The IC_50_ value of HApt conjugated DOX-loaded NPs against SKBR3 and MCF-7 cell lines was obtained to be 1.8 µg/mL and 5.5 µg/mL, respectively. The cell survival of SKBR3 cells incubated with DOX, non-conjugated DOX NPs and HAPt conjugated DOX NPs at DOX concentration of 3.6 µg/mL was decreased by 8 ± 4%, 27 ± 6% and 68 ± 6%, respectively. The cell cytotoxicity results displayed that the HAPt and DOX when co-administered by NPs showed synergistic action against HER-2 receptor overexpressed cell line.

Yang et al. developed AS1411 aptamer-conjugated NIR light sensitive nanovesicles for cancer drug delivery [122]. The MS-Au-NPs were prepared first and then aptamer AS1411 was subsequently conjugated on the surface of the gold nanorod (Au-NR-MSNPs-Apt). Prepared NPs had an average particle size of 60 nm and a silica shell size of 23 nm. In a 1 mg/mL NPs solution, the temperature and NIR dependent drug release behavior of NPs was investigated. In a temperature of 50 °C, 96% of the medication was released in 100 min. After 10 min of 0.6 W/cm2 808 NIR irradiation, drug release was 5% in 30 min and 30% in 90 min. NIH3T3 and MCF-7 cells were used to test the in vitro cellular absorption of produced NPs. MCF-7 cells had a greater cellular absorption as compared to NIH3T3 cells with Au-NR-MSNPs-Apt formulations. MCF-7 cells were also used to investigate the cytotoxicity of the produced NPs in vitro. DOX-loaded NPs had low cytotoxicity against MCF-7 cells, yet DOX-loaded NPs irradiated with NIR 808 had significantly higher cytotoxicity. With NIR 808 irradiation, the anticancer effect of AS1411 conjugated NPs was shown to be synergistically increased. The increased impact was due to the rapid drug release in the presence of NIR 808 light.

For overcoming the most prominent reason of tumor survival and reoccurence which is drug resistance, Kumar et al. developed multifunctional MSNP carriers for the co-delivery against TNBC cells [123]. The MSNP nanocarrier was synthesized and modified using poly-L-arginine, polyethylene glycol and targeted to TNBC using the AS1411 aptamer. Initially the drug doxorubicin was loaded followed by siRNA. Target specificity was confirmed by the blockade assay while assessing the cellular uptake. The results were validated in a 3D cell culture system to assess tumor penetrance. The co-delivery efficacy was evaluated on drug resistant MDA-MB-231 and a 40-fold reduction in IC_50_ values towards doxorubicin was observed when BCL-xL and BCL-2 siRNA was co-delivered. Similarly, Salve et al. developed bio-responsive MSNPs for aptamer mediated targeted delivery of paclitaxel for the treatment of ovarian cancer [124]. Using tetrasulfide derivative of silica for doping the mesoporous silica nanoparticles, the nanoparticles were biodegradable in the presence of GSH. The biodegradable MSNPs were then conjugated with MUC-1 aptamers for targeting the ovarian cancer cells. Paclitaxel was loaded on the nanocarriers and the loading was found to be ~ 40% in targeted and ~ 36% in non-targeted. The drug release was observed at pH 7.4 and pH 5.5, as well as in the presence of GSH at both pH. At physiological pH 7.4, in the presence of GSH approximately 42% release was observed, whereas, the release was ~ 28% in absence of GSH over the duration of 72 h. However, at pH 5.5 the release was ~ 93% in the presence of GSH and ~ 60% when GSH was absent, over 72 h. Cytotoxicity and apoptosis assessment was done using flow cytometry, MTT, and AO/EB staining which confirmed the higher cytotoxicity of the targeted nanoparticles as compared to non-targeted nanoparticles.

**Fig. 12:**
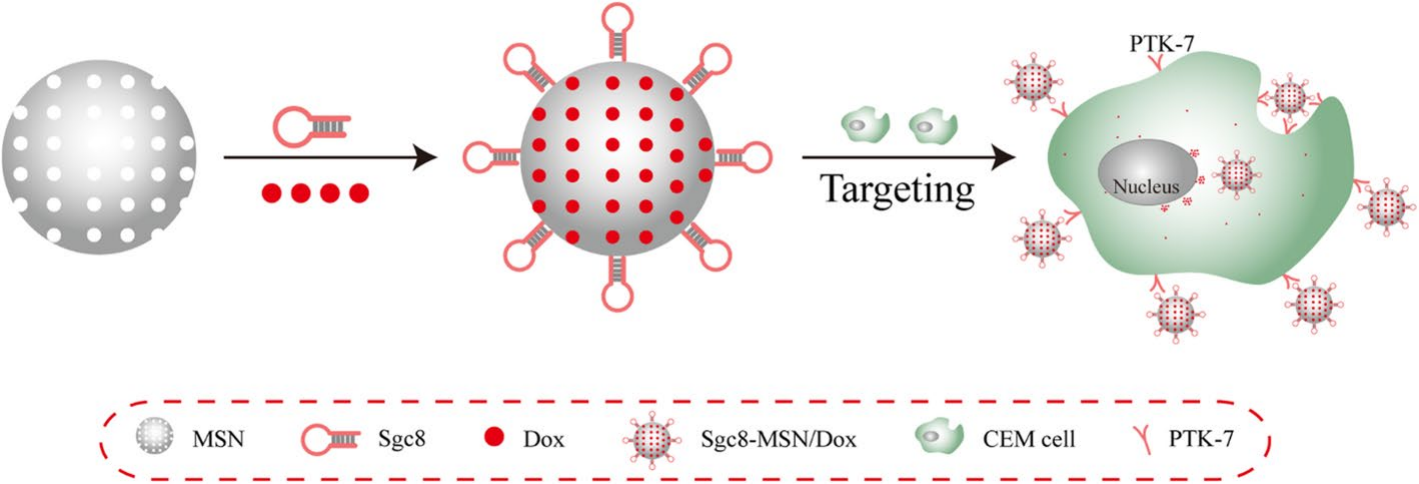
Schematic illustration of Sgc8 aptamer-modified mesoporous silica nanoparticles for an efficient cell-targeting drug delivery system. CEM cell, CCRF-CEM human acute T lymphocyte leukemia cells; Dox, doxorubicin; MSN, modified silica nanoparticle; PTK-7. Reproduced with permission from Yang et al. 2019 [125]. The figure is reproduced under Creative Commons Attribution License

Yang et al. used a nanoprecipitation approach to make Sgc8 aptamer and DOX coupled MSNPs (Sgc8-MSN-DOX) (Fig. 12) for targeted drug administration [125]. The pore size, pore volume and zeta potential of NPs were measured to be 5.23 nm, 2.51 cm^3^/g, and 33.87 mV, respectively. The drug release from NPs was 50% in 96 h at pH 7.4 and 60% in 48 h at pH 5, indicating that the produced NPs released the majority of the drug at acidic pH. CEM T lymphocyte leukemia cells and Ramos B lymphoma cells were used to test the in vitro cellular absorption of produced NPs. In Ramos B cell lines, cellular absorption of DOX-MSNPs and Sgc8-DOX-MSNPs was identical. In CEM T cells, cellular absorption of Sgc8-DOX-MSNPs was considerably greater than DOX-MSNPs. This was owing to Sgc8 Apt specificity for PTK-7 receptors found on CEM T cells, which resulted in increased uptake. Blank MSNs were tested for cytotoxicity in vitro against CEM T, Ramos, 293 T, and L-02 cell lines. Against all of the cell lines, the blank MSNPs showed no substantial cytotoxicity. The results showed that the blank MSNPs are safe to use. Sgc8-DOX-MSNPs had a substantially greater in vitro cytotoxicity against CEM T cells than DOX-MSNPs. The findings revealed that the Sgc8 Apt was more selective for PTK-7 receptor overexpressed cells and might be employed as a targeting moiety for successful anticancer therapy. AS1411 aptamer conjugated and DOX-loaded MSNPs were also reported by Li et al. for targeted medication delivery to treat breast cancer [126]. With a zeta potential of -38.5 mV, the particle size of produced NPs ranged from 50 to 150 nm with DL 4.6%. They used MCF-7 cells to test the in vitro cellular absorption of produced NPs. MCF-7 cells displayed increased DOX fluorescence in their cytoplasm due to receptor-mediated endocytosis. In LNCaP cells, cellular absorption of NPs was low. The MTT assay was used to test the anticancer activity of produced NPs against MCF-7 cells in vitro. At 1 mg/mL treatment concentrations, MCF-7 cells treated with blank aptamer coupled MSNPs demonstrated low cytotoxicity. DOX-aptamer-MSNPs and DOX-MSNPs had IC_50_ values of 990 nM and 2.4 M, respectively. The aptamer conjugated NPs had a reduced IC_50_ value, indicating that the nanocarriers enhanced targeted drug delivery with a superior anticancer impact.

Stigmergy is the strategy where there is an indirect coordination between two agents mediated by signalling a change in environment. Estepa-Fernández et al. explored this coordination strategy to evaluate its effectiveness in cancer therapy in vitro and in vivo. There are two nanoparticles involved in this strategy. Firstly, the senescence inducer drug palbociclib is loaded in MSNP and coated with heterobifunctional PEG, viz. further covalently linked with the MUC1-aptamer. Another nanoparticle loaded with senolytic navitoclax and modified using hexa-ligo-saccharide is prepared. In the first step of the strategy, the first nanoparticle targets the tumor and modifies the environment by inducting senescence. When the second group of nanoparticles come in this environment they are triggered and release their cargo to eliminate the senescent tumor cells. The conjugation of aptamer to nanoparticles was compared with a random sequence conjugated to a nanoparticle. The MUC1-aptamer conjugated nanoparticle showed 40% more uptake than random-sequence conjugated nanoparticle within 2 h of treatment. Thus, showing the efficacy of aptamer conjugation to nanoparticles for enhanced targeting and uptake by the tumor cells. When the stigmergy was evaluated between the two nanoparticles using in vivo imaging the first nanoparticle conjugated with the MUC1 aptamer showed maximum localization to the tumor. Furthermore, the second nanoparticle showed maximum accumulation and drug release in the locality of first nanoparticle. This study provides an innovative implementation for cancer therapy.

#### Bioactive/Nucleic acid/drug conjugated aptamer

Cellular transport is a key roadblock to therapeutic research and clinical use of genetic materials such as DNA, RNA, siRNA, and ribosome-inactivating proteins. Bioactive, for example, chemotherapeutic drug, siRNA, miRNA, DNA etc. have a high cytoplasmic activity for killing cancer cells. Still, their translation from bench to bedside is impeded by their undesirable characteristics, such as a short half-life, poor tumor targeting, and cell penetration. As a result, aptamer conjugated genetic materials are a novel approach for efficiently immobilizing complex molecules, such as peptides and proteins, toward biological targets. Selective vital factors affecting a nanoconjugate's ability to recognize its target include multivalency of the ligand, conformational changes during ligand binding to nanocarriers, thermodynamic and kinetic factors related to the ligand interaction with the nanocarrier, and interaction between the nanocarriers and their surroundings. The specificity and selectivity of produced nanoconjugates toward their target, as well as their potential utility in biological applications, are driven by a combination of these challenges. As a result, strategies for nanobioconjugation are required to improve the quality of next-generation nanomedicines. This section reviews molecules directly coupled with aptamers for their targeted delivery to specific cells.

MUC-1 aptamer conjugated DNA-based nanocarriers were created by Liu et al. for targeted gene delivery [127]. The MUC-1 aptamer was conjugated and the p53 gene was assembled on the triangular DNA origami with DOX folded forming a “nanokite” structure. In nanokite, the EE of DOX was found to be 50%. In MCF-7R cells, cellular absorption of MUC-1 aptamer conjugated triangular DNA origami was substantially greater than that of non-conjugated DNA origami. MCF-7R cells were utilized to investigate the anti-proliferative effectiveness, and the findings revealed that aptamer-linked DOX and p53 gene-loaded origami reduced cell viability by 20%. The tumor targeting of aptamer-attached DNA origami was assessed using fluorescence imaging, which revealed a lot of fluorescence around the tumor and tumor veins. When BALB/C mice were treated with MUC-1 aptamer coupled DNA origami, the in vivo level of the p53 gene was found 20 times higher than in the control group. Thus, the aptamer mediated uptake increased gene expression and targeting capabilities which supported in the delivery of anticancer medications to MDR cancer cells in a more efficient and focused manner. Aptamer-DNA nanoconjugates were also reported by Liu et al. for breast cancer therapy, wherein they created dual targeting DNA tetrahedron nanocarriers [128]. Using a one-pot reaction approach, they first created MUC-1 DNA tetrahedron nanocarriers and DOX-loaded MUC-1-DNA tetrahedron-AS1411 nanocarriers. The ability of produced nanocarriers to target MUC-1 overexpressed MCF-7 and MCF-7/ADR cells and normal HepG2 cells were tested. MUC-1 DNA tetrahedron-AS-1411 uptake was greater in MCF-7 and MCF-7/ADR cells, but uptake was absent in HepG2 cells. The results exhibited the target selectivity of generated nanocarriers. They assessed the cytotoxicity of these nanoconjugates in HL-7702 human normal liver cells. Even at a treatment dose of 300 nM, the placebo nanocarriers did not show cytotoxicity. Free DOX had an IC_50_ of 1.25 M against HL-7702 cells, while DOX loaded MUC-1 DNA tetrahedron-AS1411 had an IC_50_ of 10 M. The DOX-loaded MUC-1 DNA tetrahedron-AS1411 inhibited cell growth by 52% in MCF-7/ADR cells, while free DOX inhibited cell development by only 28%. The results exhibited more effectiveness of DOX aptamer nanocarrier formulations. MCF-7 and MCF-7/ADR cells were also employed to investigate the in vitro cellular absorption of nanocarriers generated. CLSM data showed that MCF-7/ADR cells absorbed DOX-loaded MUC-1 DNA tetrahedron-AS1411 at a significantly higher rate than free DOX. In MCF-7/ADR cells, they also looked at MUC-1 DNA tetrahedron-AS1411 intracellular co-localization. According to the time evolution analysis, the MUC-1 DNA tetrahedron-AS1411 entered the cytomembrane in approximately 1 h, the cytoplasm in about 3 h and the perinuclear region in 6 to 12 h. Overall, the nanocarriers were shown to be specific to MCF-7/ADR cells, and they largely delivered medicines through receptor-mediated endocytosis, and localized in nuclei via the lysosomal pathway.

**Fig. 13:**
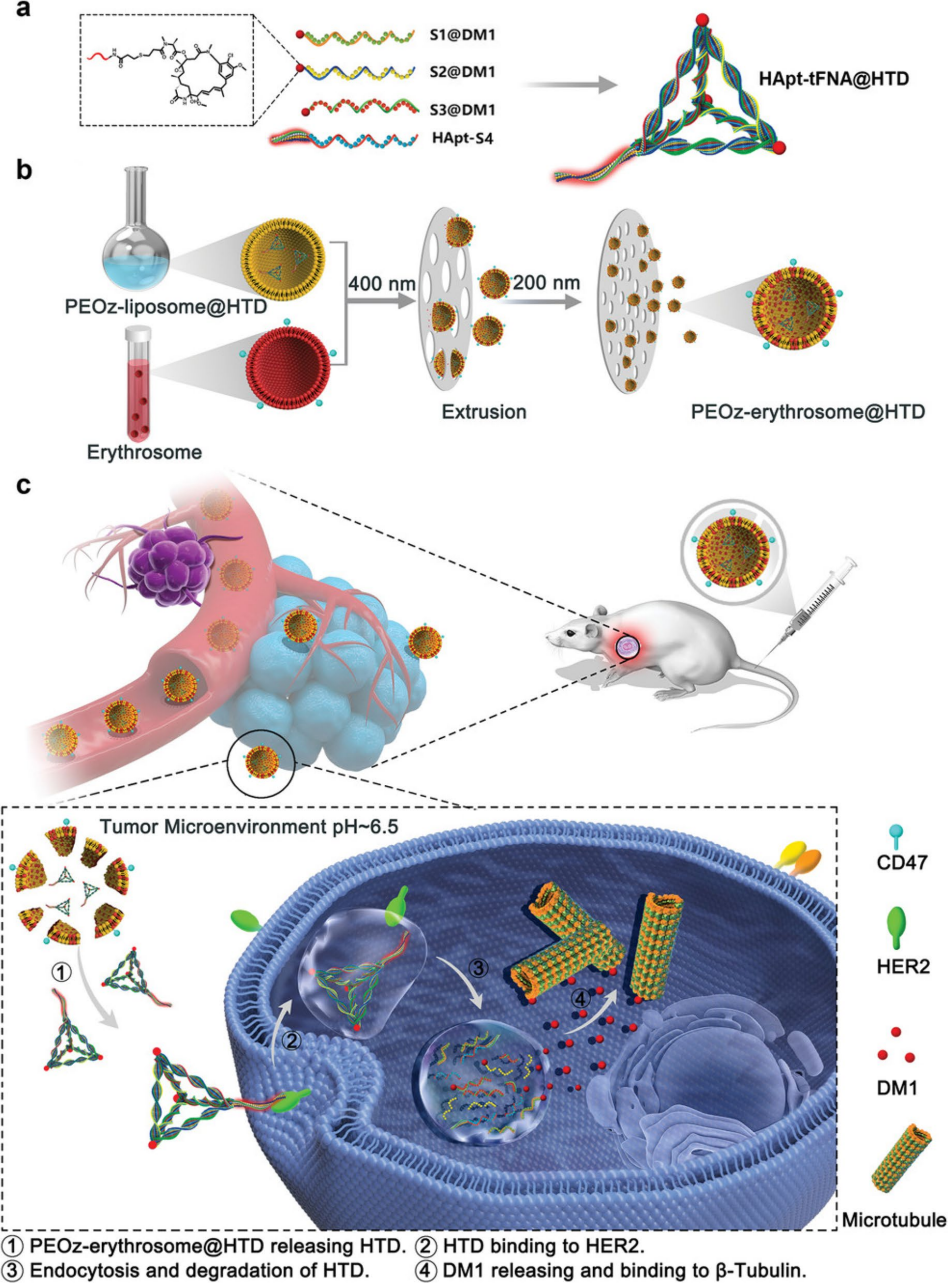
Schematic illustration of the synthesis of HApt-tFNA@DM1 (HTD), PEOz-erythrosome@HTD, and their proposed antitumor mechanism. **a** Schematic illustration of the synthetic process of HTD. b) PEOz-erythrosome@HTD was synthesized by coextrusion of PEOz-liposome@HTD and erythrosome. **c** After intravenous injection, PEOz-erythrosome@HTD was expected to target the acidic tumor microenvironment through the pH- responsive property and then release its cargo (HApt-tFNA@DM1, HTD). The HTD could bind to HER2 protein and be internalized by HER2-positive cancer cells. After the lysosomal degradation of HTD, the DM1 was released and could bind to β-tubulin. The released DM1 kills tumor cells by inhibiting the process of dissociation or assembly of microtubule (combination of β-tubulin and α-tubulin). Reproduced with permission from Ma et al. [129]. Copyright John Wiley and Sons (2022)

For the treatment of breast cancer, trastuzumab which is an HER2-antibody based anticancer therapy is most usually recommended. Similar antibody-based methods have been used to target other tumor markers. As we have seen throughout the review, aptamer can be replaced with antibodies in various therapeutic strategies. Ma et al. have engineered an aptamer-based, HER2 targeted drug delivery system [129]. A tetrahedral framework nucleic acid (tFNA) was used to increase the drug/carrier ratio and was made of ssDNA. The drug was then chemically linked with the ssDNA framework followed by HPLC to remove by-products. Furthermore, the aptamer-drug (HTD) conjugate was loaded into a hybrid of liposomes and erythrosomes for a targeted and tumor-stimulated release as shown in Fig. 13. TEM images of nanoparticles showed that the biomimetic erythrosomes disintegrated at pH 6.5 to 5.0. In vivo administration of the nanoparticles showed higher accumulation of PEOz-erythrosome NPs in the tumor and kidney in comparison with PEOz-liposome. The tumor volume was also found to be significantly decreased in the PEOz-erythrosome treatment group proving the effectivity of the novel targeted drug delivery method.

For targeted drug delivery, Taghdisi et al. produced a three-way junction pocket DNA nanostructure of DOX based on the AS1411 aptamer [130]. The average particle size of the nanostructure formed was 10.3 ± 1.3 nm. The cellular absorption of free DOX and DOX loaded DNA nanostructure was assessed in PC-3, 4T1, and CHO (Chinese Hamster Ovary cells) cell lines by detecting DOX fluorescence. The fluorescence/FL2 log intensity was studied during the internalization assay. The fluorescent pictures clearly showed that PC-3 cells absorbed DOX and DOX-loaded DNA nanostructures considerably more efficiently (1184 ± 96 and 1112 ± 121, respectively) than CHO cells and 4T1 cells. PC-3, 4T1, and CHO cells were used to test the cytotoxicity of the nanostructures generated in vitro. Their cytotoxicity data suggests that DOX had IC_50_ values of 3, 1.8, and 2.2 M in CHO, PC-3, and 4T1 cells, respectively. Cell viability in CHO, PC-3, and 4T1 cells was 95.67%, 85.79%, and 80.3 ± 1.6 percent, respectively, after the treatment with placebo DNA nanostructure. After treatment with free DOX, the vitality of CHO, PC-3, and 4T1 cells was 51.2%, 46.8%, and 47.4%, respectively. After treatment with DOX-loaded DNA nanostructure, cell viability in CHO, PC-3, and 4T1 cells was 79.6 ± 3.8%, 33.2 ± 1.5%, and 25.6 ± 2%, respectively. The produced nanostructures were less toxic to normal CHO cells and had stronger cytotoxicity against 4T1 and PC-3 cells than free DOX. The anticancer impact of nanostructures was investigated in vivo on BALB/c mice with 4T1 cell tumors. The groups were given free DOX, DOX-loaded DNA nanostructures, or PBS at a DOX concentration of 1.2 mg/kg. The tumor volume was 1049 ± 120 mm3, 845 ± 50 mm3, and 424 ± 39 mm^3^ following 20 days of therapy with PBS, free DOX, and DOX-loaded DNA nanostructures, respectively. The findings revealed that DOX-loaded DNA nanostructures have a stronger anticancer impact than free DOX in BALB/c mice with 4T1 tumors.

Pan et al. used aptamer-functionalized antisense oligonucleotides and DOX to improve therapeutic impact against drug-resistant cancer cells [131]. They used antisense DNA to deliver Bcl-2 and P-gp, as well as DOX as a chemotherapeutic drug. The zeta potential of prepared origami was found to be 12.36 ± 1.75 mV with 34.2% of DL. CLSM pictures revealed that the DOX-DNA-origami-aptamer was assimilated more in HeLa/ADR cells than standard DOX-DNA-origami, with cellular uptake increasing by 2.7 times. The endocytosis mechanism and regulation of Bcl-2 and P-gp genes in HeLa/ADR cells were also studied, and it was discovered that DOX-origami internalization was an energy-dependent process involving the lipid-raft-mediated endocytosis route. Further, the genes were significantly downregulated in cells with DOX-DNA-origami-aptamer formulations. The CCK8 assay was used to investigate how to overcome medication resistance. The HeLa/ADR cells treated with DOX, DOX-origami, DOX-DNA-origami and DOX-DNA-origami-aptamer at DOX concentration of 16 µM was 65.2%, 62.7%, 37.3% and 14.2% viable, respectively. The IC_50_ value of DOX, DOX-origami, DOX-DNA-origami and DOX-DNA-origami-aptamer at DOX in HeLa/ADR cells was found to be 20.1 ± 0.3, 19.8 ± 0.3, 11.6 ± 0.1 and 6.2 ± 0.1 µM, respectively. The IC_50_ value of DOX, DOX-origami, DOX-DNA-origami and DOX-DNA-origami-aptamer at DOX in MCF-7/ADR cells was exhibited as 38.5 ± 0.6, 37.4 ± 0.5, 20.6 ± 0.2, and 12.1 ± 0.1 µM, respectively. The results clearly prove that aptamer conjugation, as well as DNA origami, can treat drug-resistant cancer.

**Fig. 14:**
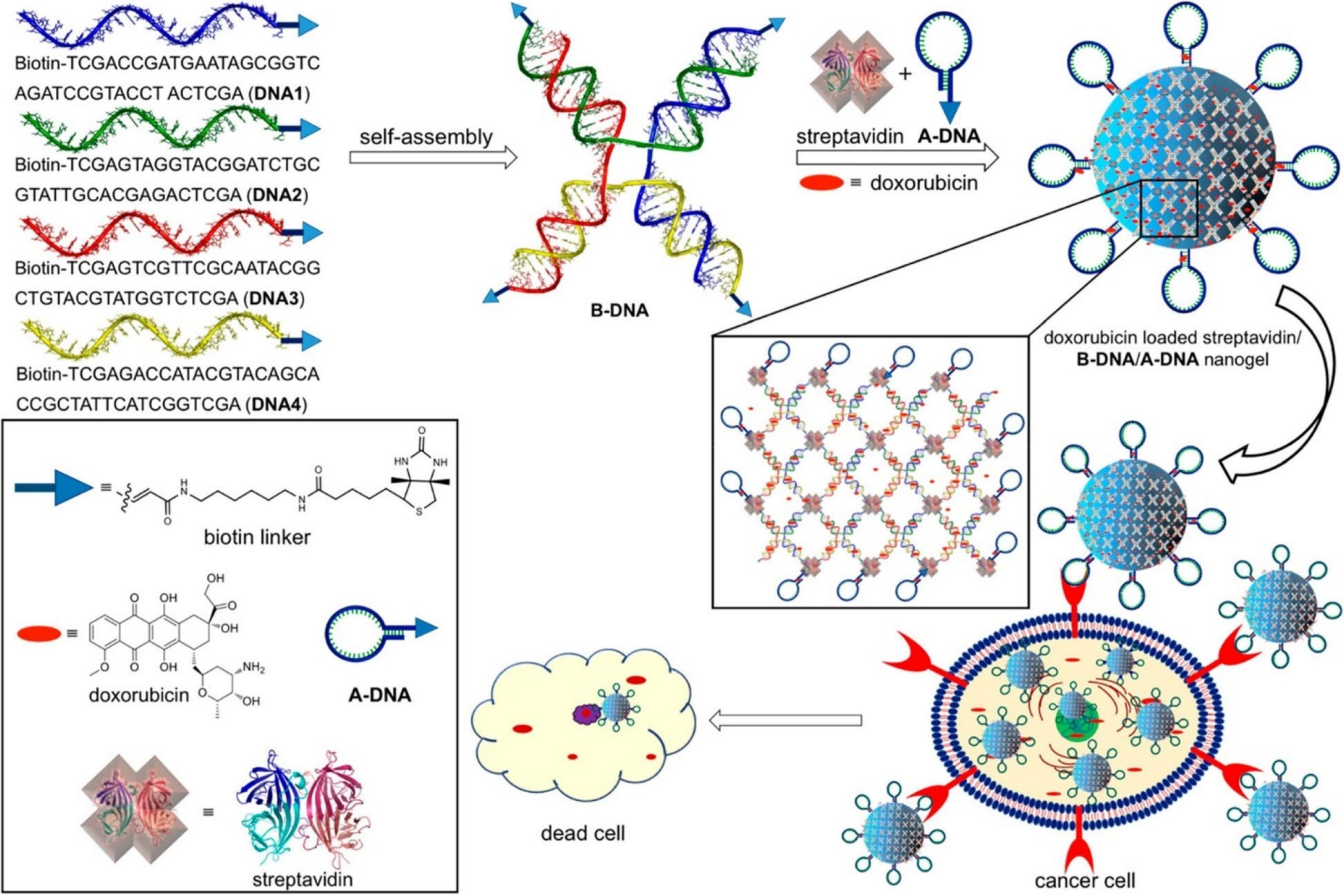
Design of doxorubicin-loaded, aptamer-decorated nanogels through multiple streptavidin–biotin interactions by the simultaneous assembly of B-DNA, streptavidin, A-DNA, and doxorubicin. Diffusion of doxorubicin-loaded nanogels inside the cell through receptor-mediated endocytosis and delivery of doxorubicin and apoptosis are depicted. Chemical structures of the biotin derivative, doxorubicin, and streptavidin are also given (left bottom corner). Reproduced with permission from Thelu et al. [134]. Copyright American Chemical Society (2019)

Xiang et al. produced a CSC marker EpCAM aptamer-DOX complex for the efficient killing of chemotherapy-resistant cancer stem cells [132]. In PBS (pH of 5.0) at 72 h extended time, drug release from the aptamer-DOX combination was found at 89%. The aptamer-DOX combination has a binding affinity of 16.08 ± 4.83 nM for HT29 cells and more than 1000 nM for KEK293T cells. In HT29 cells, cellular absorption of the aptamer-DOX complex was 2.5 × greater than that of free DOX. In HT29 cells, the self-renewal ability of CSCs was investigated after treatment with aptamer-DOX or free DOX. The HT29 cells were planted in stem cell culture before being treated with 1 µM free DOX or an aptamer-DOX equivalent. When compared to free DOX treatment, aptamer-DOX treatment reduces CSC cell frequency by 16.7%. The in vivo data suggest that, in the first, second, and third rounds, the tumor spheroid was reduced by 10.4, 12.37, and 170 times, respectively, after therapy with aptamer DOX. The reduction in tumorsphere with aptamer conjugated drug treatment was also investigated in SKOV-3 and T47D cells. In HT29 cells injected NOD/SCID mice, the in vivo decrease in CSC frequency to develop tumor in immunocompromised mice after therapy with aptamer-DOX was investigated. The results showed that when 1 × 10^4^ cells treated with aptamer-DOX were transplanted into NOD/SCID mice, no tumors formed. The findings showed that using a CSC-targeting aptamer, a conventional chemotherapeutic agent may be converted into a CSC-killer to overcome drug resistance in solid tumors. Mei et al. created DOX DNA nanoflowers (NFs) as a tailored drug delivery device for MDR cancer treatment [133]. K562/MDR cells were targeted with a KK1B10 aptamer, whereas MCF-7/MDR cells were targeted with a sgc8 aptamer. NFs have a monodispersed spherical form with a diameter of 200 nm, as seen in SEM pictures. In KK1B10-NFs, DOX loading was found to be 71.4 percent w/w. The release of drugs from NFs in vitro was examined at pH 7.4, 5.0, and 9, and it was discovered that drug release was faster at pH 5.0 than at pH 9 and 7.4. K562, K562/MDR, and Ramos cells were used to test the in vitro cellular absorption of KK1B10-NFs. The uptake of KK1B10-NFs in K562 and K562/MDR cells was much greater, but it was limited in Ramos cells. Similarly, the uptake of sgc8-NFs in MCF-7 and MCF-7/MDR cells was significantly higher, but it was low in Ramos cells. The drug content inside the K562/MDR cells steadily decreased following treatment with free DOX, but it remained constant after treatment with KK1B10-NFs. MCF-7/ADR cells treated with sgc8-NFs showed the same outcomes. NF-Dox generates considerable cytotoxicity in both the targeted chemosensitive cells and the MDR cells, but not in non-target cells, preventing MDR while also reducing side effects. After treatment with free DOX and sgc8-NFs, the IC_50_ values in MCF-7/ADR cells were found at 200 µM and 42.7 µM, respectively. Likewise, after treatment with free DOX and KK1B10-NFs, the IC_50_ values in K562/ADR cells were found at 50 µM and 13.2 µM, respectively. The significantly low IC_50_ values of aptamer conjugated NFs demonstrated that these formulations could be employed to treat MDR tumors effectively. Thelu et al. developed DOX-B-DNA/streptavidin decorated with A DNA protein complex to form hybrid nanogel for cancer therapeutic targeting (Fig. 14) [134]. The particle size and zeta potential of the prepared complex were found to be 68–225 nm and − 41.982 mV, respectively. In CCRF-CEM, HeLa, and Ramos cells, the nanogel complex was efficiently absorbed and equally dispersed throughout all cell lines. FACS techniques were used to evaluate the permeability of Cy-tagged B-DNA/streptavidin/A-DNA in the three cell lines. Cy-tagged B-DNA/streptavidin/A-DNA was shown to be taken up by CCRF-CEM and HeLa cells because of the presence of PTK-7 receptors, but not by Ramos cells. The viable cell count of CCRF-CEM, HeLa, and Ramos cells after treatment with free DOX was found to be around 35%, 40%, and 50%, whereas for DOX-B-DNA/streptavidin/A-DNA the viability was 37%, 42%, and 85%, respectively in different cell lines. According to the findings, aptamer linked nanogels are more cytotoxic to CCRF-CEM and HeLa cells than free DOX, but not to PTK-7 receptor-negative Ramos cells.

**Fig. 15:**
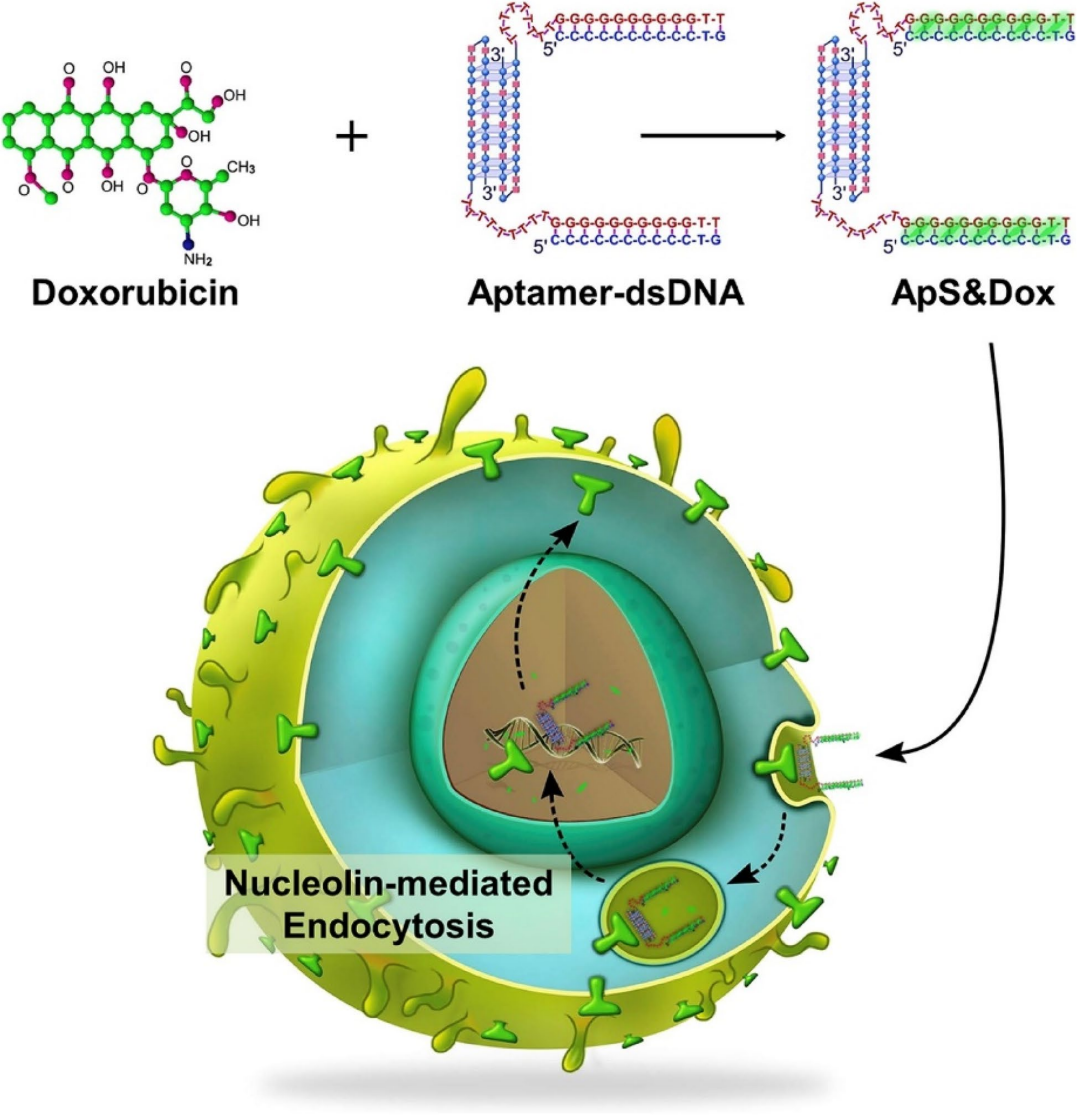
Schematic illustration of the targeted delivery of doxorubicin using a functionalized aptamer to circumvent multidrug resistance. Doxorubicin (Dox) intercalates into the GC-rich dsDNA of Aptamer-dsDNA (ApS) to form ApS&Dox nanoparticles. Aptamer binds specifically to nucleolin (green) on the cell surface. The nucleolin/ApS&Dox complex is endocytosed and delivered to the nucleus, where doxorubicin intercalates with the genomic DNA. (For interpretation of the references to color in this figure legend, the reader is referred to the web version of this article). Reproduced with permission from Liu et al. [135]. Copyright Elsevier (2016)

Liu et al. prepared multifunctional aptamer-based NPs of DOX (Fig. 15) for treatment of drug resistant breast cancer [135]. The oligonucleotide sequences they created were S2, S4, S6, S6-1, S6-2, S6-3, S6-4, S8, S10, S20, ApS6, and ApS10. Nanostructures were created when the designed ApS6 or ApS10 oligonucleotides annealed together. The complex of ApS and DOX at a molar ratio of 1:1, designated ApS6-DOX and ApS10-DOX, were utilized for further experiments among the produced complexes. The mean particle size of ApS6-DOX and ApS10-DOX was found to be 6.1 ± 0.7 nm and 7.4 ± 0.4 nm, respectively. The in vitro cytotoxicity of prepared NPs was evaluated against MCF-7R, MCF-7S and L02 cells. The IC_50_ value of ApS10-DOX, ApS6-DOX, aptamer and free DOX against MCF-7R cells was reported to be 2.45, 9.44, 19, > 30.00 µM, respectively. The enhanced antiproliferative effect was also observed in A549 cells. The cell cycle data clearly revealed that when MCF-7R cells were treated with ApS10-DOX NPs, they accumulated substantially more in S phase than when they were treated with free DOX. This indicated the more effectiveness of ApS10-DOX NPs in MCF-7R cells. The in vivo antitumor effect of prepared NPs was studied in MCF-7R tumor xenografted mice. The tumor inhibition rate of the group treated with ApS10-Dox NPs was obtained at 88.2% and groups treated with Aptamer and free DOX alone were obtained at 66.7% and 21.6%, respectively. The greater tumor inhibition after treatment with ApS10-Dox NPs exhibited the enhanced anticancer effect against MCF-7R drug-resistant cancer.

## Limitations and overcoming methods

The paper focused on recent advancements in aptamer-attached nanocarriers and their use in cancer diagnostics and therapy. The fast deterioration of aptamers (specifically RNA aptamers) through nucleases in biological conditions, and predominantly in blood, is a major issue that limits their hands-on applicability. Current anti-cancer nanocarriers for therapy and diagnostics are largely antibody based and are gullible to the drawbacks associated with the use of antibodies. Replacing aptamers with antibodies for target specificity allows versatile chemical synthesis, lowered immunogenic response, and higher stability. These benefits are further complemented by the conjugation of the aptamers with nanoparticles. When aptamers are conjugated to the nanoparticle, one end of the aptamer is conjugated to the nanoparticle and is therefore unavailable for exonuclease degradation, reducing susceptibility of aptamers. After SELEX method, nucleotides already contained in aptamers may be altered; but, the addition of extra-functional moieties in this scenario could alter its affinity and specificity [136]. However, certain changes can make aptamers more resistant to nucleases without compromising their ability to attach to target molecules. Renal filtration removes most of the free aptamers from circulation owing to low molecular weight, making their therapeutic usage more challenging. Combining aptamers with a PEG (10–20 kDa) is the most popular solution to this problem [137]. This technique is currently being employed to prolong the circulation duration of bioactives such as proteins, peptides and oligonucleotides. Pharmacokinetic characteristics of a drug (for example, action duration) are critical in its therapeutic application. Antidotes against aptamers can be made by synthesizing a complementary oligonucleotide, which is one option. When an aptamer is hybridized with antidote, it changes shape and loses its ability to bind the target molecule completely. Aspects of aptamer interaction with intracellular targets provide a few challenges as well. However, there have been substantial recent breakthroughs in aptamer intracellular delivery.

## Conclusion and future perspectives

Aptamer, a unique and specialized tool towards a target, is gaining popularity in cancer detection and treatment. Recent advances in this promising field of aptamer, such as new methods in aptamer screening utilizing the SELEX approach, aptamer modification, and aptamer applications, particularly for cancer treatment, were reviewed in this manuscript. Aptamers have already been successful in the sensitive and selective identification of certain cancer cell types or tissues as effective targeting ligands. Bioconjugates that combine nanomaterials with aptamers will speed up the development of effective cancer treatment techniques. Because they seldom break down in biological systems, the combination of organic and inorganic nanomaterials with an aptamer moiety offers significant advantages when used as drug carriers. The influencing properties of aptamer coupled nanoconjugates in the field of cancer nanomedicine have developed hopeful objectives for cancer subjugation. A number of SELEX-based aptamers show clinical potential as independent treatments or in conjunction with other traditional chemotherapeutics to treat various cancers. Furthermore, the new drug delivery techniques improved aptamers' target-specific therapeutic potential. However, when coupled with aptamers, which have unique structural characteristics, it may be possible to develop novel cancer treatment methods.

Despite tremendous progress towards tumor application research, aptamers still require improvements in medicine loading quantity, targeting accuracy, circulation time, and affinity, among other things. It is essential to have a deeper knowledge of the interactions between aptamers and medicines, as well as how aptamers influence and alter nanocarrier properties. For remediating the drawbacks associated with aptamers, conjugation with nanoparticles increases the drug loading ability and circulation time of the nanocarrier. Thus, both individual components viz. nanoparticles and aptamers when combined have a synergistic effect and functionally complement each other. Throughout the article we saw novel strategies such stigmergy and use of metal organic frameworks (MOFs) utilizing nanoparticle-aptamer conjugates for anti-cancer therapy. Moreover, aptamers have been conjugated to all types of nanoparticles including most of organic and inorganic nanoparticles. We expect aptamers to play a more important role in cancer therapeutic applications in the future, as SELEX technology and various synthesis techniques develop and get widely implemented. Based on the potential multimodal theranostic nanoplatforms and a growing demand for effective cancer therapy, we will see continuous incremental advancement of aptamer-conjugated nanomaterials for treating cancer in the near future. Upcoming research will focus on developing multimodal aptamer conjugated nanomaterials that would incorporate both diagnostic and therapeutic components to address concerns such as multiple-drug resistance, with the hope of optimizing treatment outcomes and lowering costs.

## Data Availability

Not applicable.
